# A hierarchical model for strategic and operational planning in blood transportation with drones

**DOI:** 10.1371/journal.pone.0291352

**Published:** 2023-09-14

**Authors:** Amirali Amirsahami, Farnaz Barzinpour, Mir Saman Pishvaee

**Affiliations:** School of Industrial Engineering, Iran University of Science and Technology, Tehran, Iran; Cyprus International University Faculty of Engineering: Uluslararasi Kibris Universitesi Muhendislik Fakultesi, TURKEY

## Abstract

Blood transportation is a critical aspect of the healthcare systems, ensuring whole blood and blood products are delivered to patients in a timely and efficient manner. However, transportation of blood and other medical supplies can be challenging, especially in urban areas with limited infrastructure and heavy traffic. Drones have become increasingly important in recent years as a means of delivering medical supplies, including blood, due to their ability to provide fast, reliable, and cost-effective transportation. This study proposes two mathematical programming models in the hierarchical structure to improve decision-making for strategic and operational planning in the blood supply chain network. The limited information available in strategic planning presents risks to the blood supply chain, making it imperative to address uncertainties. To tackle this challenge, a novel approach called Scenario-based Robust Bi-objective Optimization has been proposed. The first model employs this approach to efficiently handle demand uncertainty by simultaneously maximizing the covered demand and minimizing costs. The model is subsequently solved using the augmented ε-constraint method. The second model is a routing-scheduling operational model that aims to minimize the sum of operations time, taking into account time windows for blood collection centers and hospitals. The developed hierarchical model is implemented in a three-level supply chain of Tehran province under three crisis scenarios in different parts. The findings and analysis of this implementation suggest that it is beneficial to set up drone stations in cost-effective and central locations to avoid costly network design. Furthermore, utilizing the minimum number of feasible drones enhances operational time and results in cost savings and increased efficiency. Overall, this study highlights the potential of using drones for blood transportation in urban settings, which can have significant implications for improving the quality of healthcare delivery.

## 1. Introduction

Blood is a vital biological fluid that is crucial in human health and medical practice. The high demand for whole blood or its products, such as red blood cells, platelets, plasma, and cryoprecipitate, has spurred significant research in the field of the blood supply chain [1]. There are an estimated 118.5 million blood donations collected worldwide every year, so efficient and effective transportation methods are crucial for global healthcare [2]. Considering emergency circumstances within this supply chain, it is essential to design the blood transportation network in a manner that allows for the delivery and utilization of blood as quickly as possible. Consequently, the utilization of drones for the expeditious transportation of blood and related products has been recommended in recent medical and transportation studies [3, 4]. Unmanned aerial vehicles (UAV), commonly referred to as drones, are essentially aircraft that operate without a pilot on board and can be programmed to function autonomously or controlled manually to perform a variety of missions. In recent years, drones have gained many applications, including healthcare, commercial, and humanitarian logistics. Among the rationales for drone deployment are expedited cargo transportation, high and consistent velocity, the need for no road infrastructure, and not depending on road congestion [5].

Generally, medical logistics applications of drones rely on time-critical products such as patient diagnostics, pharmacy supplies, and blood transfusions, which, in most cases, are low-volume [6–8]. The use of drones for transporting these critical items is particularly useful in both urban and remote areas where traditional transportation methods are limited, providing a fast, reliable, and cost-effective means of transportation for medical supplies and equipment. Overall, the use of drones in medical logistics has great potential to transform healthcare services in both urban and rural areas.

The transportation of blood and its products is a crucial and intricate operation that directly impacts human lives. Due to the perishable nature of these products, it is imperative to thoroughly evaluate any new transportation methods for the blood supply chain, taking into account various aspects of feasibility. In recent medical research, the use of drones for blood transportation has shown promising results, further highlighting the potential of this innovative approach. For example, Amukele et al. [3] conducted tests to assess the quality of blood products such as red blood cells, platelets, and plasma, transported via drones. Remarkably, their findings revealed no adverse effects on the quality of these products, establishing the feasibility of drone-based blood transportation. Capitalizing on this potential, startups in the drone logistics industry have embarked on drone projects aimed at revolutionizing medical logistics, with a particular focus on blood transportation. Noteworthy examples include Wingcopter in Tanzania, which successfully transports blood, Flirtey in the United States, delivering defibrillators, and Matternet in Switzerland, facilitating blood transportation [9–11]. Among these initiatives, Zipline’s project in Rwanda stands out as a resounding success. In 2019 alone, this endeavor accounted for 20% of blood distribution outside the country’s capital [12]. As emphasized by Nisingizwe et al. [13], drones have not only demonstrated increased speed in blood transportation but have also played a crucial role in reducing blood shortages by an impressive 67% in Rwanda.

Blood transportation is a vital aspect of the healthcare industry that requires careful planning and logistics to ensure the safe and timely delivery of blood products. Traditional transportation modes face significant challenges in urban areas, such as traffic congestion, limited infrastructure, and difficulty accessing certain areas, which can delay the delivery of blood products, risking their quality and integrity. To address these challenges, drones have emerged as a potential solution to provide rapid, cost-effective, and safe delivery of blood products in the blood supply chain. Despite their potential, the use of drones in blood transportation has received limited attention from Operations Research researchers. However, the few scholars who have studied this field have proposed models for decision-making at different levels, defining the problem and making assumptions in their articles. In this context, this study aims to advance the use of drones in the blood supply chain through the development of two mathematical programming models. These models address decision-making at both strategic and operational levels and have been implemented on the three-level supply chain of Tehran province, including Blood Collection Centers (BCC), Blood Transfusion Centers (BTC), and hospitals. At the strategic level, a newly proposed scenario-based Robust Bi-objective Optimization approach considers the uncertainty of transportation flows and addresses critical decisions, including the location and allocation of drone stations, as well as fleet selection. This approach is presented in the context of mathematical programming under uncertainty, offering an innovative solution to the field. The model aims to minimize costs and maximize demand coverage simultaneously, using the augmented ε-constraint method. The proposed model with the selected solution method, enabling decision-makers to easily choose their preferred decision based on their preferences, which is challenging to achieve using a scenario-based robust optimization approach, while also assigning uncovered demand to the existing transportation system, considering the potential violation of constraints, rather than employing a two-stage stochastic programming approach. At the operational level, the presented mathematical programming model focuses on decisions such as routing and scheduling of drone operations under time windows to minimize the total operation time. Both models aim to optimize the blood supply chain and make it more efficient, cost-effective, and reliable. The potential of drones in blood transportation is significant, and the models developed in this study can be used as effective tools to optimize the blood supply chain and ensure the safe and timely delivery of blood products in urban areas and beyond.

The contributions of this study to respond to the research gaps found are summarized below:

- A hierarchical model is introduced that takes into account the complete structure of the blood supply chain network in the urban environment for both strategic and operational planning.
- The most important decisions involved in strategic and operational planning are given attention, including location-allocation and routing-scheduling respectively.
- Fleet management is considered in strategic and operational planning through decision-making regarding fleet selection, allocation, and sizing.
- A Scenario-based Robust Bi-objective Optimization approach is presented for strategic planning as a novel approach in the field of mathematical programming under uncertainty, to deal with demand uncertainty.

- A novel mixed-integer linear programming model that addresses the routing and scheduling of drones to minimize the sum of operations time is presented.
- New assumptions are considered compared to the assumptions of previous routing studies in this field, such as considering the hard time windows and also the multi-trip nature of drones.

The following article is structured as follows. Section 2 provides an overview of relevant literature. Section 3 defines the problem and presents two mathematical programming models. In Sections 4 and 5, a case study of Tehran’s blood supply chain is presented, along with analyses and managerial insights based on the results generated by the models. Finally, in Sections 6 and 7, conclusions are drawn, and potential future directions for research are suggested.

## 2. Literature review

Advances in drone technology have ushered in a new era of possibilities in delivery and transportation, with far-reaching implications across diverse sectors, including healthcare and e-commerce [14]. Drones have demonstrated exceptional capabilities in efficiently transporting a wide range of cargo, reshaping logistics operations across industries. Particularly notable is the integration of drones into emergency and healthcare services, a sector where their potential to revolutionize critical operations has captured significant attention.

Within the realm of emergency and healthcare services, drones have exhibited remarkable aptitude in transporting vital biological samples, notably blood products. Preliminary findings underscore their ability to maintain controlled temperatures over short distances, preserving the accuracy of laboratory tests [3, 15–18]. Such efficiency holds the promise of substantial reductions in delivery times and costs, effectively elevating service quality. As a result, researchers are vigorously pursuing full integration of drones into the emergency and healthcare landscape, focusing on pivotal areas such as blood transportation.

Contrasting this backdrop, a comprehensive exploration of the blood supply chain spanning over five decades has unveiled the intricate dimensions encompassing storage, inventory management, queuing, distribution, and transportation [19–21]. This complexity has prompted scholars to deploy a repertoire of mathematical programming models and algorithmic strategies, ranging from exact methods to heuristics and metaheuristics [1, 22, 23]. However, while these endeavors have considerably enriched the field of Operations Research, the transformative potential of integrating drones into blood supply chain optimization remains largely untapped. A closer inspection of Table 1 reveals a distinct emphasis on blood transportation within emergency and healthcare services, underscoring its pivotal role in optimizing logistical efficiency. Strategically, these studies have harnessed mathematical optimization tools to guide decision-making and planning. Proposed models have factored in the constraints and attributes of drone-based transportation systems and blood supply chain networks, enabling more pragmatic decisions for real-world execution. These considerations extend to drone flight range, a fundamental constraint in delivery operations, coupled with limited drone capacity that curtails the carriage of sizable loads over long distances. Consequently, as Table 1 illustrates, a majority of investigations, particularly in the strategic domain, have approached drones as single-visit entities.

**Table 1:** Features of related studies in drone-based emergency and healthcare services.

										Strategic Decision			Operational Decisions								
Authors	Year	Application	Model	# Of drones	Capacity	Range	# Of trip	Visits per trip	Time window	Fleet selection	Facility location	Facility allocation	Fleet sizing	Fleet allocation	Routing	Scheduling	# Of objectives	Objective	Uncertain parameter	Mathematical programming model	Solution method
Kim et al. [24]	2017	O	H	M	✓	✓	1	1			✓						1	Min-Cost		IP	Heuristics
				M	✓	✓	1	M					✓		✓		1	Min-Cost		MIP	
Chauhan et al. [25]	2019	O	I	M		✓	1	1			✓	✓		✓			1	Max-Cov		IP	Heuristics
Ozkan & Atli [26]	2021	O	I	M	✓	✓	1	M							✓		1	Min-Distance		MIP	Exact
Ghelichi et al. [27]	2021	O	I	M	✓	✓	1	M	✓		✓	✓				✓	1	Min-Time		IP	Exact
Jung & Kim [28]	2022	O	I	M		✓	1	1						✓		✓	1	Max-Cov		RO	Exact
Ghelichi et al. [29]	2022	O	H	M		✓	1	1			✓	✓					1	Min-Time + Max-Cov	Demand Location	SP	Heuristics
				M		✓	1	1								✓	1			IP	
Chauhan et al. [30]	2022	O	I	M		✓	1	1			✓	✓					1	Max-Cov	Travel Time	RO	Exact
Shi et al. [31]	2022	O	I	M	✓	✓	1	M	✓		✓	✓	✓		✓		2	Min-Cost/ Min-Time		MIP	Heuristics
Park et al. [32]	2023	O	I	M		✓	1	M			✓	✓		✓			1	Min-Cost	Demand	RO	Heuristics
Wen et al. [33]	2016	B	I	M	✓	✓	1	M							✓		2	Min # of drones/ Min-Distance		IP	Heuristics
Dhote & Limbourg [34]	2020	B	I	1		✓	1	1			✓						1	Min-Cost		IP	Exact
Otero et al. [35]	2020	B	I	M	✓	✓	1	1			✓	✓					1	Min-Cost		MIP	Exact
Ghasemi et al. [36]	2021	B	I	M	✓	✓	1	M			✓	✓			✓		3	Max-Cov / Max weight of located/ Min-Cost		MIP	Exact
Rashidzadeh et al. [37]	2021	B	I	M		✓	1	1			✓	✓		✓			3	Min-Cost & Min-Emi & Max-Cov		IP	Exact
Hou et al. [38]	2021	B	I	M	✓	✓	1	M			✓	✓					1	Min-Cost	Demand	DRO	Exact
Al-Rabiaah et al. [39]	2023	B	I	M	✓	✓	1	M						✓	✓		2	Min-Distance/ Min # of drones		IP	Heuristics
Ozkan [1]	2023	B	I	M	✓	✓	1	M							✓		2	Min # of drones/ Min-Distance		IP	Heuristics
The Current Study	2023	B	H	M	✓	✓	1	1		✓	✓	✓					2	Min-Cost/ Max-Cov	Demand	SRBO	Exact
				M	✓	✓	M	M	✓				✓	✓	✓	✓	1	Min-Time		MIP	Exact

O: Other applications studies, B: Blood transportation application studies. I: Integrated, H: Hierarchical, M: Multiple. Min-Cost: Costs minimization, Max-Cov: Coverage maximization, Min-Distance: Minimize the total travel distance, Min-Time: Time minimization, Min # of drones: Minimize the number of drones, Min-Emi: Emission minimization. IP: Integer Programming, MIP: Mixed-Integer Programming, RO: Robust Optimization, SP: Stochastic Programming, DRO: Distributionally Robust Optimization, SRBO: Scenario-based Robust Bi-objective Optimization. Heuristics: category of heuristic and metaheuristic solution methods, Exact: category of Exact solution methods and commercial solvers.

In the context of the current absence of drones within emergency and healthcare logistics networks, these studies hold implications for network redesign, underlining the significance of strategic and operational decisions. Consequently, research efforts have concentrated on these dual decision-making tiers. Strategic research in blood transportation revolves around objectives like cost minimization [34, 35, 37, 38] or coverage maximization [36, 37]. This strategic decision-making also extends to the positioning of drone facilities, encompassing drone stations, charging stations [24, 30, 34, 35, 38, 40–42], and distribution centers [37], all pivotal within logistics networks. Conversely, operational-level studies primarily center on drone routing, an indispensable logistical facet in any supply chain network. Fleet management encompasses decisions such as fleet size, allocation, and selection, aspects that a handful of drone delivery studies have addressed, including Rashidzadeh et al. [37] and Al-Rabiaah et al. [39], who focused on fleet allocation. In the landscape of healthcare logistics, strategic and operational decisions assume substantial significance, particularly amidst uncertainties. Researchers have introduced uncertain variables, deploying techniques like stochastic programming [40] and robust optimization [30, 38, 41] to fortify system resilience. However, the handling of uncertainties within the context of drone-based blood transportation remains limited. For instance, Rezaei et al. [43] formulated uncertain blood demand as a two-stage stochastic problem, while Hou et al. [38] proposed a data-driven two-stage stochastic optimization model employing the Wasserstein distance to minimize network costs while adhering to flow, capacity, and energy constraints.

In recent years, the confluence of operations research and logistics expertise has generated an array of drone applications for diverse cargo types. Although the majority of drone delivery literature centers on e-commerce logistics, a substantial body of work has also emerged within emergency and healthcare services, as depicted in Table 1. Thus, for a truly impactful integration of drones into blood supply chain enhancement, it becomes imperative not only to assimilate existing research within this domain but also to delve into related studies within emergency and healthcare services. Moreover, insights from domains like e-commerce logistics, sharing operational parallels with drone operations, can offer valuable perspectives. Echoing earlier sentiments, addressing operational decisions alongside strategic considerations forms the cornerstone of blood transportation network redesign. Notably, studies by Ghaseami et al. [36] and Rashidzadeh et al. [37] seamlessly incorporate both dimensions. Similarly, research on emergency and healthcare services predominantly underscores these two facets in conjunction with blood transportation [27, 31]. However, several strategic and operational aspects remain uncharted within these studies. Among these underexplored facets, fleet management decisions–both strategic and operational–stand out, particularly in the context of drone delivery for emergency and healthcare services. Notably, while e-commerce research gravitates towards routing and scheduling decisions [14, 44, 45], the emergency and healthcare services literature seldom delves into these areas. Instead, strategic decisions, such as facility location and allocation, garner heightened attention. Notably, disparities emerge due to unique constraints, exemplified by the heightened focus on strict delivery deadlines in e-commerce logistics, contrasting with scarce research within emergency and healthcare services concerning these constraints [46, 47]. The landscape of optimization models within transportation network redesign spans diverse objective functions, as aptly illustrated in Table 1. Evidently, research predominantly centers around cost minimization or demand coverage maximization. Curiously, despite the availability of multiple multi-objective models in blood supply chain research, the exploration of integrating these two objectives remains conspicuously absent. Conversely, objective functions focused on minimizing time-related parameters, such as makespan, extensively examined within e-commerce research, find minimal representation within the realm of blood transportation [48, 49]. As certain investigations have embraced such objective functions within emergency and healthcare services, the research gap within blood transport becomes pronounced, urging further exploration and consideration. The disparities delineating research in emergency and healthcare services from that in e-commerce underpin a landscape rife with gaps arising from environmental and priority disparities. This divergence, however, offers a fertile ground for innovation within both domains. However, certain facets, including fleet management, have remained overlooked across both arenas, resulting in research voids extending beyond these sectors. Foremost among these gaps is the paucity of attention to uncertainties, a quintessential branch of mathematical optimization scarcely applied in the context of drone delivery research. A dearth of investigations leverages these approaches to model and dissect the inherent uncertainties of drone-based systems. Upon scrutiny of Table 1, a common thread emerges: the majority of researchers present integrated models, tailored to their specific research problem assumptions. This inclination has steered their focus towards decision-making encompassing both operational and strategic spectrums. Notably, both Shi et al. [31] and Park et al. [32] adeptly navigate both strategic and operational tiers through integrated models. Contrasting this approach, Kim et al. [24] and Ghelichi et al. [29] adopt a separate hierarchical modeling for these tiers, a perspective that diverges from the common practice within the field of blood transportation. While the application of hierarchical modeling facilitates a comprehensive exploration of decision-making within a given problem, its rarity in the blood transportation domain highlights the potential for more exhaustive exploration by researchers confronting a specific research challenge.

It is imperative to acknowledge that drone delivery, while novel in logistics literature, is distinguished by its distinct focus on technological advancements. This trajectory has inevitably imposed constraints within the research process. Notably, recent endeavors by operations research scholars have propelled the integration of drones into various delivery and transportation applications. Mathematical models like IP, MIP, and RO have been introduced to grapple with these challenges, relying on either exact or heuristic methods dictated by problem nature. These studies have not only advanced theoretical frameworks within operations research but have also spurred the growth of businesses, industries, and healthcare systems.

This article introduces new mathematical models to address a gap in existing research related to planning for both strategic and operational aspects. Specifically, we present a unique approach called Scenario-based Robust Bi-objective Optimization, which deals with mathematical problems when there’s uncertainty involved. This approach aims to achieve two goals: reducing average costs and minimizing the percentage of incomplete drone deliveries in blood supply chains, considering the uncertainties that arise in transportation. To solve this approach, we use a method called augmented ε-constraint, which helps us make decisions about where to place facilities, how to allocate resources, and which fleet to use. Building on the insights from the strategic model and using an extended version of the traveling repairman problem [50], we also develop an operational model for routing and scheduling. This model takes into account the importance of specific time windows, which is particularly relevant in time-sensitive healthcare operations within cities. The main objective of this operational model is to minimize the overall time taken for operations, which is crucial to meet strict time constraints in healthcare settings. Additionally, this operational model provides valuable insights into short-term decisions about how to allocate and size the fleet, addressing an aspect that hasn’t been widely explored in previous research.

## 3. Methodology

The speed of transportation in healthcare logistics directly affects human lives. Therefore, the decision has been made in this study to use drones to increase the speed of blood and blood products transportation in the forward logistics. Since the standard distribution of blood requires attention to numerous factors, it is assumed that the drones used are equipped with devices that can transport blood under suitable conditions in terms of temperature, stability, and other factors. As previously mentioned, drones are a new transportation trend, and they are intended to be added to an ongoing blood supply chain network. Redesigning such a network requires planning and decision-making at strategic, tactical, and operational levels, and this research mainly focuses on two levels: the strategic and the operational.

**Fig 1:**
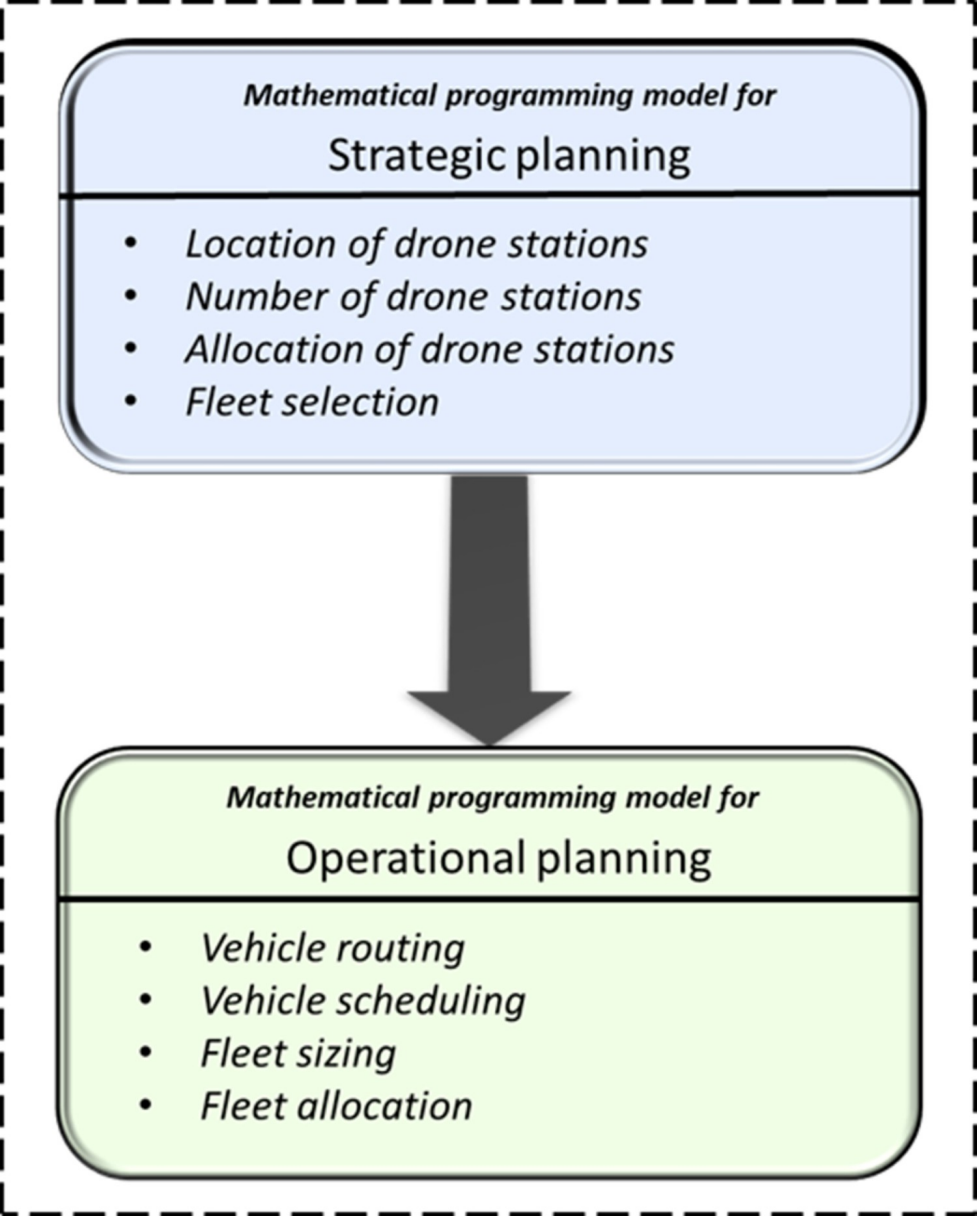
The proposed hierarchical model.

As depicted in (Fig 1), the mathematical programming model for strategic planning and its corresponding solution method are utilized to derive four decisions, including the location of drone stations, determination of the number of drone stations, allocation of drone stations, and fleet selection. These decisions are then considered as parameters for the mathematical programming model for operational planning, which is responsible determining operational decisions, such as drone routing, scheduling, fleet sizing, and fleet allocation.

The research methodology employed in this study is a quantitative approach that utilizes mathematical programming. In the following sections, namely 3.1 and 3.2, the problem description and modeling approach are explained in detail. Subsequently, in sections 3.3 and 3.4, the strategic and operational models are presented.

### 3.1. Problem description at the strategic level

**Fig 2:**
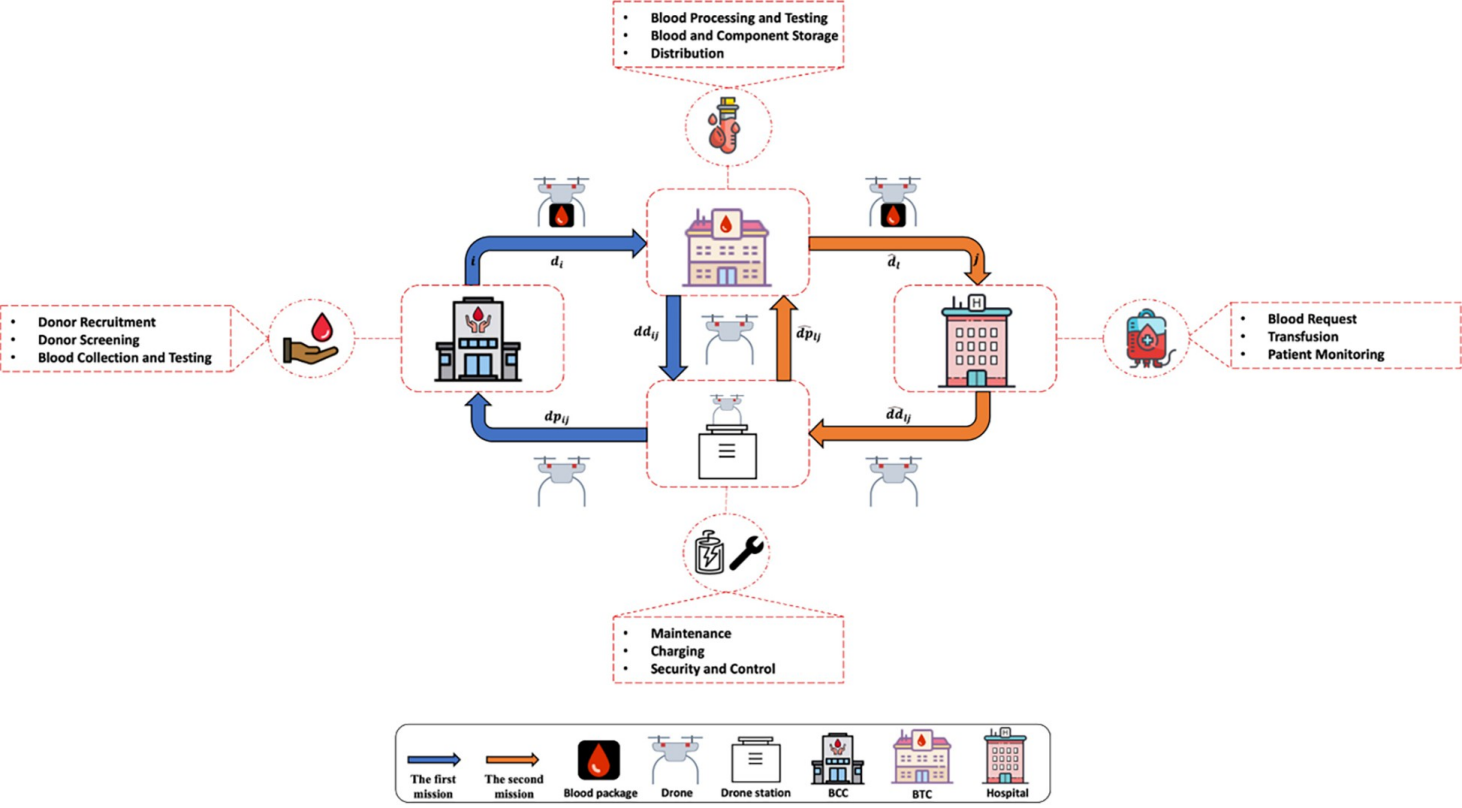
Drone missions considered in the blood supply chain network (in the strategic model). Republished from icons8.com under a CC BY license, with permission from Icons8, original copyright 2023.

The blood supply chain network, illustrated in (Fig 2), operates through three interconnected levels: BCCs, BTCs, and hospitals. In this network, the flow of items varies across the layers. Within the first layer, primarily comprising whole blood, donations constitute a significant portion of the items. The transportation of these items is facilitated by various types of ground vehicles, following a predetermined plan at specific time intervals. Moving to the second layer, a diverse range of blood products flows through the system. Here, transportation of items occurs in response to hospital requests, catering to the needs of the blood bank and emergency situations. Similarly, a variety of ground vehicles are employed for this purpose. The blood supply chain encompasses distinct responsibilities for each level. BCCs are primarily responsible for blood collection and conducting basic tests. BTCs, on the other hand, handle additional testing, processing donated blood into various components, managing inventory, and distributing blood products. Hospitals play a vital role by placing orders for specific blood products from BTCs and administering transfusions to patients.

This study aims to determine the proportion of blood transport currently reliant on ground vehicles that can be effectively delegated to drones. Consequently, in the strategic part of this study, it is assumed that a drone, after taking off from its drone station, can have two types of missions: 1) collection from BCCs and delivery to BTCs (first layer) 2) collection from BTCs to hospitals (second layer). Drones, given their characteristics, can cover a certain distance and naturally, if a drone is within its flight range, it can perform multiple missions consecutively. However, due to the focus of this research on the strategic level of planning and also the difference in demand distribution between the two missions (layers), only the two mentioned missions are considered in this study. After completing each mission, the drone returns to its base station to recharge and/or repair. These two missions are schematically shown in (Fig 2) (the notations in this figure are referred to in Table 2).

**Table 2:** Notations used in the mathematical model for strategic planning.

***Sets***	
*J* = {1,…,*m*}	Set of Candidate drone station locations
*I* = {1,…,*n*}	Set of arcs that related to the flows between BCCs and BTCs
*L* = {1,…,*s*}	Set of arcs that related to the flows between BTCs and hospitals
*Ω* = {1,…,*k*}	Set of all scenarios
***Indices***	
*j*∈*J*	Index of candidate drone station locations
*i*∈*I*	Index of arcs that related to the flows between BCCs and BTCs
*l*∈*L*	Index of arcs that related to the flows between BTCs and hospitals
*θ*∈*Ω*	Index of scenarios
***Parameters***	
${\tilde{f}}_{i\theta }$	Number of required drone flights in arc *i* under scenario *θ* (uncertain)
dp_ij_	Distance between drone station *j* and BCC in arc *i*
*d*_*i*_	Distance between BCC and BTC in arc *i*
dd_ij_	Distance between BTC in arc *i* and drone station j
${\tilde{\hat{f}}}_{l\theta }$	Number of required drone flights in arc *l* under scenario *θ* (uncertain)
${\hat{dp}}_{lj}$	Distance between drone station *j* and blood transfer center in arc *l*
${\hat{d}}_{l}$	Distance between BTC and hospital in arc *l*
${\hat{dd}}_{lj}$	Distance between hospital in arc *l* and drone station *j*
*b*_*j*_	Cost of establishing a drone station at candidate location *j*
*α*	Cost of empty drone flight per kilometer
*β*	Cost of loaded drone flight per kilometer
*A*	Drone flight range in kilometers
*π*_*θ*_	Probability of scenario *θ* occurring
*M*	A sufficiently large positive number
***Decision variables***	
*x*_*ijθ*_	If arc i is assigned to drone station *j* under scenario *θ*, one; otherwise, zero (second-stage)
${\hat{x}}_{ij\theta }$	If arc l is assigned to drone station *j* under scenario *θ*, one; otherwise, zero (second-stage)
*w*_*j*_	If a drone station is established at candidate location *j*, one; otherwise, zero (first-stage)
*ε*_*θ*_	Total number of unperformed drone flights between BCCs and BTCs under scenario *θ*
${\hat{\varepsilon }}_{\theta }$	Total number of unperformed drone flights between BTCs and hospitals under scenario *θ*

#### 3.1.1 Scenario-based robust bi-objective optimization

Due to the long-term nature of strategic planning and decision-making, the available information is often limited, which leads to uncertainties that can put the blood supply chain network at risk if not considered. In order to address various uncertainties, several approaches have been proposed under the general titles of stochastic programming, robust optimization, and fuzzy programming in the field of mathematical optimization. Therefore, in order to cope with uncertainty in the demand parameter in the investigated application of this research and to further develop the field of stochastic programming and robust optimization, a new approach titled Scenario-based Robust Bi-objective Optimization has been introduced.

To gain a better understanding of the approach of Scenario-based Robust Bi-objective Optimization in dealing with uncertainty, the following text provides a detailed description of its structure and underlying logic, along with a comparison to two well-known stochastic programming approaches, namely Two-stage Stochastic Programming and Scenario-based Robust Optimization. It should be noted that all the notations used in this section are specific to this particular section and are purely mathematical symbols for representing the logic of Stochastic Programming approaches to dealing with uncertainty. In fact, these symbols do not have any specific interpretation and are general representations that can be completely independent of the notations that will be introduced in subsequent sections and if necessary, reference will be made to this section in other sections. In Model (1), we assume that "*X*" and "*Y*" are two non-negative variables and "*C*_1_^*T*^" and "*C*_2_^*T*^" represent the transposed vectors of cost coefficients for "*X*" and "*Y*" in the objective function. Meanwhile, the variable "z" represents the value of the objective function. "*H*_1_" and "*H*_2_", on the other hand, represent the technological coefficients of "*X*" and "*Y*", respectively. Additionally, there is a randomness uncertainty associated with a value known as "$\tilde{B}$" on the right-hand side [51].

$$\begin{array}{l} Min\;z={C_{1}}^{T}X+{C_{2}}^{T}Y\\ s.t.\\ H_{1}X+H_{2}Y\geq \tilde{B}\\ X,Y\geq 0 \end{array}$$ (1)

Stochastic Programming is a conventional strategy employed to tackle uncertainty arising from randomness. It treats uncertain parameters as random variables following known probability distributions. However, in the context of complex real-world supply chains, incorporating continuous distribution functions can result in computationally intractable problems. To address this challenge, discrete scenarios are often utilized to describe uncertain parameters, with each scenario assigned a probability of occurrence [52, 53]. Hence, we assume that "$\tilde{B}$" has occurred based on the presented scenarios labeled as "*θ*∈*Ω*", leading us to update its notation from "$\tilde{B}$" to "*B*_*θ*_". As shown in Model (2), the Two-stage Stochastic Programming model is formulated with two variables: the first-stage variable (here and now) and the second-stage variable (wait and see). The second-stage variable, denoted as "*X*", assumes different values depending on the scenario, and its value is influenced by the first-stage variable, "*Y*", which remains constant across all scenarios [54]. In the context of the problem discussed in this study, the first-stage variable represents the location variable of drone stations, while the second-stage variable represents the allocation variable. Consequently, the value of the allocation variable relies on the location of the drone station, which remains consistent across all scenarios.

$$\begin{array}{l} Min\;z_{\theta }={C_{1}}^{T}X_{\theta }+{C_{2}}^{T}Y\\ s.t.\\ H_{1}X_{\theta }+H_{2}Y\geq B_{\theta }\;\theta \in \Omega \\ X_{\theta },Y\geq 0\;\theta \in \Omega \end{array}$$ (2)

As outlined in Mulvey’s publication [55] titled Robust Optimization of Large-Scale Systems, Model (3), that is also known as Scenario-based Robust Optimization model includes a *Φ*_*θ*_ as a variable that is designed to absorb the effects of any constraint violation that may happen. However, to discourage such violations, a penalty coefficient "*ψ*" is introduced. The objective function of the model is divided into three parts. The first part aims to minimize the expected value of the main objective function across different scenarios. The second part accounts for the deviation of the objective function value in each scenario from the expected value and helps to ensure optimality robustness, and the third part controls feasibility robustness. It should be noted that "*θ*" is equivalent to "*θ′*" and is considered in this way to prevent any mistakes in calculating the expected value within the second term. The importance coefficients of the first two terms, denoted as *λ* and 1−*λ*, should be between 0 and 1. The importance coefficients, along with the penalty for violating the constraint in the third term, need to be adjusted in a way that not only implements the decision maker’s preferences but also allows for effective interpretation.

$$\begin{array}{l} Min\;\underset{\theta }{\lambda E}(z_{\theta })+(1-\lambda )\underset{\theta }{E}\left({\left(z_{\theta }-\underset{\theta^{\prime }}{E}(z_{\theta^{\prime }})\right)}^{2}\right)+\psi \left(\underset{\theta }{E}(\Phi_{\theta }^{2})\right)\\ s.t.\\ H_{1}X_{\theta }+H_{2}Y\geq B_{\theta }-\Phi_{\theta }\;\theta \in \Omega \\ X_{\theta },Y,\Phi_{\theta }\geq 0\;\theta \in \Omega \end{array}$$ (3)

Now, the Model (4) called the Scenario-based Robust Bi-objective Optimization model presented in this study simultaneously seeks to minimize the expected value of the main objective function under different scenarios and also minimize the expected value of constraint violations, without the need for parameter tuning and in a transparent manner. In the specific context explored in this article, which focuses on the blood supply chain, the primary objective is to minimize the average cost of the system. Meanwhile, the secondary objective aims to minimize the average unmet demand.


$$\begin{array}{l} Min\;\underset{\theta }{E}(z_{\theta })\\ Min\;\underset{\theta }{E}(\Phi_{\theta })\\ s.t.\\ H_{1}X_{\theta }+H_{2}Y\geq B_{\theta }-\Phi_{\theta }\;\theta \in \Omega \\ X_{\theta },Y,\Phi_{\theta }\geq 0\;\theta \in \Omega \end{array}$$ (4)

### 3.2. Problem description at the operational level

**Fig 3:**
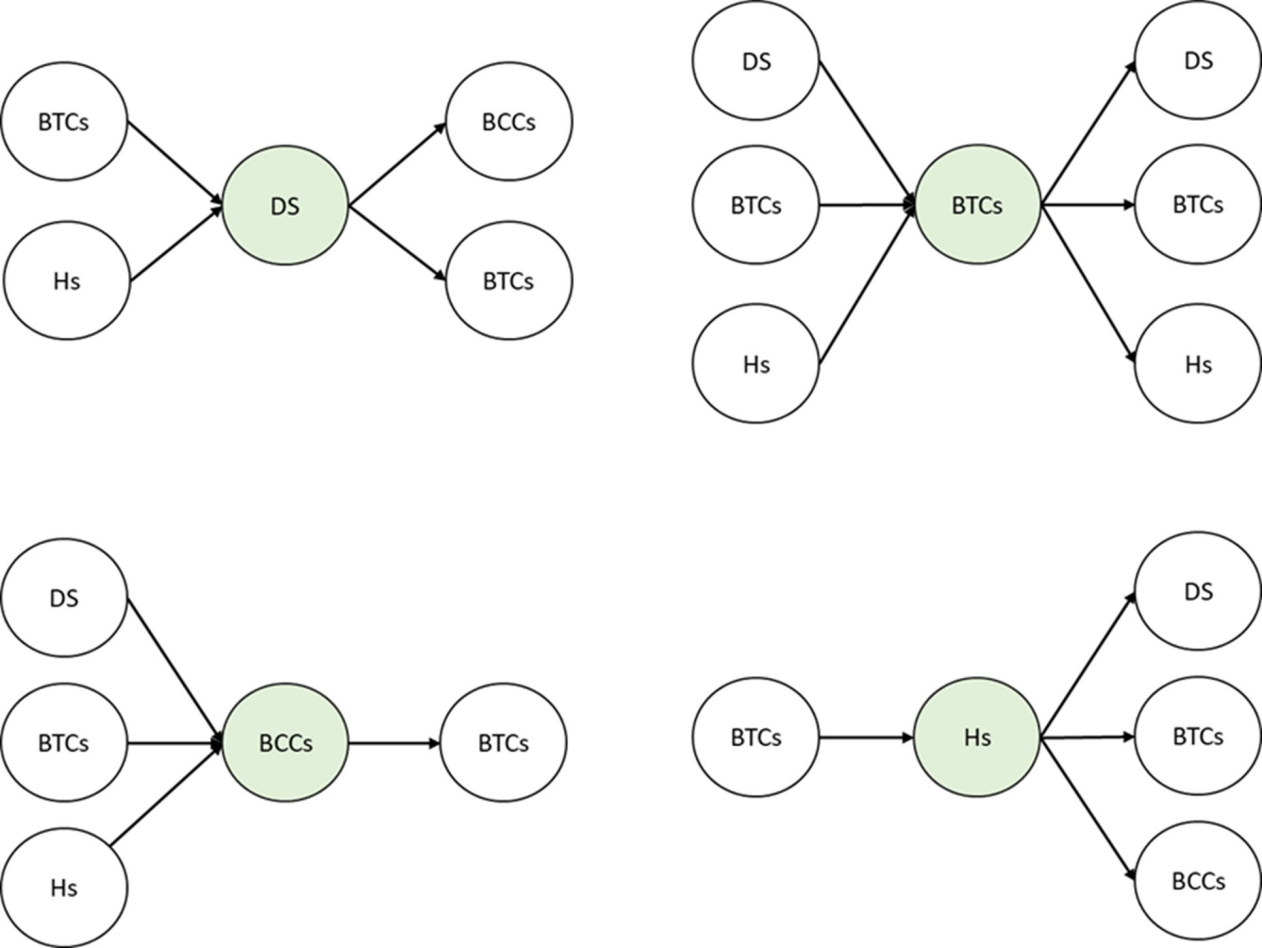
Possible movements between nodes of the blood transportation network (in the operational model). DS: Drone Station, BCCs: Blood Collection Centers, BTCs: Blood Transfusion Centers, Hs: Hospitals.

The focus should shift to short-term planning once strategic decisions have been made. The supply chain network, which is the output of strategic planning, plays a crucial role in these decisions, which deal with the daily operation of a system. In this study, we assume that we are at the beginning of the planning horizon, at time 0, and we want to perform operational planning for a drone station that has been selected and a flow has been assigned to it at the strategic decision-making level. According to the status of BTCs, drones must take a specific number of flights from BCCs to BTCs to transport donated blood, according to their capacity and the designated time window. Additionally, drones must travel to hospitals during a specified time interval in order to supply the desired blood products from BTCs at a specific number of times. These operations should be performed in a way that minimizes the total time that each drone spends on each of its trips, and these trips should be carried out without exceeding the drone’s flight range or violating the available time windows. One of the key differences between operational and strategic modeling is the possible missions of each drone on each trip. Since it is precisely determined in the short-term planning horizon how many units of blood and blood products should be transported between levels within a specified interval, more missions can be considered for mathematical modeling than in the strategic phase. Therefore, the following assumptions have been considered in the drone routing:

- On each trip, a drone must start and end its route at a drone station.
- Each node in each trip can be visited only once.

- Based on the functional differences among the nodes in the blood transportation network, specific limitations need to be considered for each node, as illustrated in (Fig 3) These limitations are due to the fact that drones are required to deliver blood from BCCs to BTCs and from BTCs to hospitals, as in strategic planning. At the operational level, drones can address the remaining missions after completing each of these missions. There is no special achievement for the blood transportation network in direct movements from drone stations to hospitals, so such movements should be removed from the list of possible movements. Taking into account the complexity of this issue, it is important to note the importance of addressing scheduling decisions along with routing decisions in order to address this issue effectively.

For operational planning, these conditions are assumed in order to make the problem as real as possible, and reducing the sum of operations time in the supply chain network so the patient can receive medical care more quickly.

### 3.3. Mathematical model for strategic planning

This section introduces indices, sets, parameters, and variables that are elaborated upon in Table 2 before discussing the presented model. All the notations introduced in this section are only relevant to this section, and if necessary, reference will be made to this section in other sections. It should be noted that paying attention to (Fig 2) provides a better understanding of the modeled system.


$$Min\;\sum_{\theta =1}^{k}\pi_{\theta }(\alpha (\sum_{i=1}^{n}\sum_{j=1}^{m}f_{i\theta }dp_{ij}x_{ij\theta }+\sum_{l=1}^{s}\sum_{j=1}^{m}{\hat{f}}_{l\theta }{\hat{dp}}_{lj}{\hat{x}}_{lj\theta })+\beta (\sum_{i=1}^{n}\sum_{j=1}^{m}f_{i\theta }d_{i}x_{ij\theta }+\sum_{l=1}^{s}\sum_{j=1}^{m}{\hat{f}}_{l\theta }{\hat{d}}_{l}{\hat{x}}_{lj\theta })+\alpha (\sum_{i=1}^{n}\sum_{j=1}^{m}f_{i\theta }dd_{ij}x_{ij\theta }+\sum_{l=1}^{s}\sum_{j=1}^{m}{\hat{f}}_{l\theta }{\hat{dd}}_{lj}{\hat{x}}_{lj\theta })+\sum_{j=1}^{m}b_{j}w_{j})$$ (5)

$$Min\;\sum_{\theta =1}^{k}\pi_{\theta }(\frac{\varepsilon_{\theta }+{\hat{\varepsilon }}_{\theta }}{\sum_{i=1}^{n}f_{i\theta }+\sum_{i=1}^{s}{\hat{f}}_{l\theta }})$$ (6)

***s*. *t*.**

$$\sum_{j=1}^{m}x_{ij}\leq 1\;\forall i\in I$$ (7)

$$\sum_{j=1}^{m}{\hat{x}}_{lj}\leq 1\;\forall l\in J$$ (8)

$$\sum_{i=1}^{n}\sum_{j=1}^{m}f_{i\theta }x_{ij\theta }\geq \sum_{i=1}^{n}f_{i\theta }-\varepsilon_{\theta }\;\forall \theta \in \Omega$$ (9)

$$\sum_{l=1}^{s}\sum_{j=1}^{m}{\hat{f}}_{l\theta }{\hat{x}}_{lj\theta }\geq \sum_{l=1}^{s}{\hat{f}}_{l\theta }-{\hat{\varepsilon }}_{\theta }\;\forall \theta \in \Omega$$ (10)

$$\sum_{i=1}^{n}x_{ij\theta }\leq w_{j}M\;\forall \theta \in \Omega \;\forall j\in J$$ (11)

$$\sum_{l=1}^{s}{\hat{x}}_{lj\theta }\leq w_{j}M\;\forall \theta \in \Omega \;\forall j\in J$$ (12)

$$x_{ij\theta }(dp_{ij}+d_{i}+dd_{ij})\leq A\;\forall \theta \in \Omega \;\forall j\in J\;\forall i\in N$$ (13)

$${\hat{x}}_{lj\theta }({\hat{dp}}_{lj}+{\hat{d}}_{l}+{\hat{dd}}_{lj})\leq A\;\forall \theta \in \Omega \;\forall j\in J\;\forall l\in L$$ (14)

$$w_{j},x_{ij\theta },{\hat{x}}_{lj\theta }\in \{0,1\}\;\forall \theta \in \Omega \;\forall i\in I\;\forall j\in J\;\forall l\in L$$ (15)

$$\varepsilon_{\theta },{\hat{\varepsilon }}_{\theta }\geq 0\;\forall \theta \in \Omega$$ (16)

The objective function (5) minimizes the average cost of the system, which includes the cost of operating drones empty and loaded, and the cost of setting up drone stations. Objective function (6) minimizes the average uncovered demand by the drone system. Constraints (7) and (8) ensure that each arc is covered by at most one drone station. Constraints (9) and (10) emphasize that the covered demand in each scenario should be at least equal to the total demand, considering the possibility of violating the constraint. Constraints (11) and (12) prevent assigning flows to non-existent drone stations. Constraints (13) and (14) ensure that a mission assigned to a station is within the flight range of its drones. Types and ranges of decision variables are shown in constraints (15) and (16).

Multi-objective optimization problems pose a challenge as there is no single optimal solution that can effectively optimize the mathematical model for all objectives, primarily due to conflicting objectives [56–58]. Therefore, it becomes necessary to find efficient solutions, known as Pareto-optimal solutions. While the ε-constraint method is commonly employed for this purpose, it does not guarantee the efficiency of the obtained solutions. To address this issue, Mavrotas [59] introduced the augmented ε-constraint method in 2009.

In this particular study, the augmented ε-constraint method has been utilized to tackle the multi-objective optimization problem. By applying this method, a set of efficient solutions is derived, offering the decision-maker a range of choices based on their preferred trade-off between the system’s average cost and unmet demand. The utilization of this method facilitates the identification of Pareto-optimal solutions that represent the optimal compromise between the conflicting objectives.

### 3.4. Mathematical model for operational planning

First, in Table 3, a concise explanation of indices, sets, parameters, and variables used is provided. This is followed by the presentation of the model. All the notations introduced in this section are only relevant to this section, and if necessary, reference will be made to this section in other sections. It should be noted that if you pay close attention to Figs 2 and 3, the model becomes more understandable.

**Table 3:** Notations used in the mathematical model for operational planning.

***Sets***	
*V* = {0,…,*m*+1}	Set of all nodes (0 represents the drone station and m+1 is an imaginary node which is actually the same as 0)
*V*′ = {1,…,*m*}⊂*V*	Set of all nodes except the drone station
*D*⊂*V*′	Set of BCCs
*N*⊂*V*′	Set of BTCs
*H*⊂*V*′	Set of Hospitals
*U*	Set of drones
*R*	Set of drone trips
***Indices***	
*i*,*j*∈*V*	Index of all nodes
*u*∈*U*	Index of drones
*r*∈*R*	Index of drone trips
***Parameters***	
*dis*_*ij*_	Travel time between nodes *i* and *j* by drone (minute)
*M*	A sufficiently large positive number
*L*	Flight range limit of drone (km)
*s*_*j*_	Service time at node *j* (minute)
*q*_*ij*_	Number of required flights from BCC point *i* to BTC point *j*
${\hat{q}}_{ij}$	Number of required flights from BTC point *i* to hospital point *j*
*l*_*j*_	Deadline for traveling to the BCC point *j* (minute)
${\hat{l}}_{j}$	Deadline for traveling to hospital point *j* (minute)
***Decision variables***	
$x_{ij}^{ur}$	1, if drone *u* uses arc *(i*, *j)* to move from node i to node *j* in *r*’th trip; otherwise, 0.
*a*_*j*_	Arrival time of drone at node *j*
*d*_*i*_	Departure time of drone at node *i*


$$Min\;\sum_{u\in U}\sum_{r\in R}(a_{m+1}^{ur}-d_{0}^{ur})$$ (17)

***s*. *t*.**

$$\sum_{j\in V\backslash \{0\}}x_{ij}^{ur}\leq 1\;\forall i\in V\backslash \{m+1\}\forall u\in U\;\forall r\in R$$ (18)

$$\sum_{i\in V^{\prime }}x_{im+1}^{ur}\leq 1\;\forall u\in U\;\forall r\in R$$ (19)

$$\sum_{i\in V}\sum_{j\in V}x_{ij}^{ur}\leq M\sum_{j\in V^{\prime }}x_{0,j}^{ur}\;\forall u\in U\;\forall r\in R$$ (20)

$$\sum_{i\in V}\sum_{j\in V}x_{ij}^{ur}\leq M\sum_{i\in V^{\prime }}x_{i,m+1}^{ur}\;\forall u\in U\;\forall r\in R$$ (21)

$$\sum_{j\in V}x_{ij}^{ur}=\sum_{j\in V}x_{ji}^{ur}\;\forall i\in V^{\prime }\forall u\in U\;\forall r\in R$$ (22)

$$\sum_{i\in V}\sum_{\begin{array}{c} j\in V\\ i=j \end{array}}x_{ij}^{ur}=x_{0,m+1}^{ur}=\sum_{j\in V}x_{m+1,j}^{ur}=\sum_{i\in V}x_{i,0}^{ur}=\sum_{j\in H}x_{0,j}^{ur}=\sum_{i\in D}x_{i,m+1}^{ur}=\sum_{i\in H}\sum_{j\in H}x_{ij}^{ur}=\sum_{i\in D}\sum_{j\in D}x_{ij}^{ur}=\sum_{i\in D}\sum_{j\in H}x_{ij}^{ur}=0\;\forall u\in U\;\forall r\in R$$ (23)

$$\sum_{u\in U}\sum_{r\in R}x_{ij}^{u,r}=q_{ij}\;\forall i\in D\;\forall j\in N$$ (24)

$$\sum_{u\in U}\sum_{r\in R}x_{ij}^{u,r}={\hat{q}}_{ij}\;\forall i\in N\;\forall j\in H$$ (25)

$$\sum_{i\in V\backslash \{m+1\}}\sum_{j\in V\backslash \{0\}}x_{ij}^{u,r+1}\leq M\sum_{i\in V\backslash \{m+1\}}\sum_{j\in V\backslash \{0\}}x_{ij}^{ur}\;\forall u\in U\;\forall r\in R$$ (26)

$$d_{i}^{u,r}+dis_{ij}-(1-x_{ij}^{ur})M\leq a_{j}^{ur}\;\forall i\in V\backslash \{m+1\}\;\forall j\in V\backslash \{0\}\;\forall u\in U\;\forall r\in R\;i\neq j$$ (27)

$$a_{m+1}^{ur}-(1-\sum_{j\in V^{\prime }}x_{0j}^{u,r+1})M\leq d_{0}^{u,r+1}\;\forall u\in U\;\forall r\in R\;i\neq j$$ (28)

$$a_{j}^{ur}+s_{j}(\sum_{i\in V\backslash \{m+1\}}x_{ij}^{ur})M\leq d_{j}^{ur}\;\forall j\in V^{\prime }\;\forall u\in U\;\forall r\in R\;i\neq j$$ (29)

$$a_{j}^{ur}\leq M\sum_{i\in V\backslash \{m+1\}}x_{ij}^{ur}\;\forall i\in V\backslash \{0\}\;\forall u\in U\;\forall r\in R$$ (30)

$$s_{i}^{ur}\leq M\sum_{j\in V\backslash \{0\}}x_{ij}^{ur}\;\forall j\in V\backslash \{m+1\}\;\forall u\in U\;\forall r\in R$$ (31)

$$a_{j}^{ur}\leq l_{j}\;\forall j\in D\;\forall u\in U\;\forall r\in R$$ (32)

$$a_{j}^{ur}\leq {\hat{l}}_{j}\;\forall j\in H\;\forall u\in U\;\forall r\in R$$ (33)

$$a_{m+1}^{ur}-d_{0}^{ur}\leq L\;\forall u\in U\;\forall r\in R$$ (34)

$$a_{j}^{ur}\geq 0\;\forall j\in V\backslash \{0\}\;\forall u\in U\;\forall r\in R$$ (35)

$$d_{i}^{ur}\geq 0\;\forall i\in V\backslash \{m+1\}\;\forall u\in U\;\forall r\in R$$ (36)

The objective function (17) seeks to minimize the sum of time that each drone spends traveling during each trip in order to perform the operation. Constraints (18) and (19) guarantee that each node in each trip can be visited only once. Each drone trip must begin and end at a drone station as prescribed by constraints (20) and (21). Constraint (22) represent flow continuity in the drone’s route. Constraint (23) prevents forbidden movements between the nodes of the blood transfusion network. It is enforced by constraints (24) and (25) that there must be a certain number of trips made between levels in the blood transportation network. The constraint (26) ensures that drone trips are conducted in the correct order. In each of the nodes, the four constraints (27) to (31) calculate the departure and arrival times of drones. The hard time window constraint is applied through two constraints (32) and (33). The constraint (34) prohibits drones from flying longer than the maximum flight range. As a final point, the decision variables are defined by constraints (35) and (36).

## 4. Results

The blood supply chain network in Tehran province needs to serve at least 160 hospitals, which has led to a significant demand for blood in the hospitals of this province due to the high floating population [60]. There are fourteen BCCs in Tehran province, and the blood donated from these centers is transported to the only BTC in the province, namely the Vesal center, on a predetermined schedule (usually every three hours), and then transferred to hospitals across the province based on their needs.

### 4.1. Strategic planning

**Fig 4:**
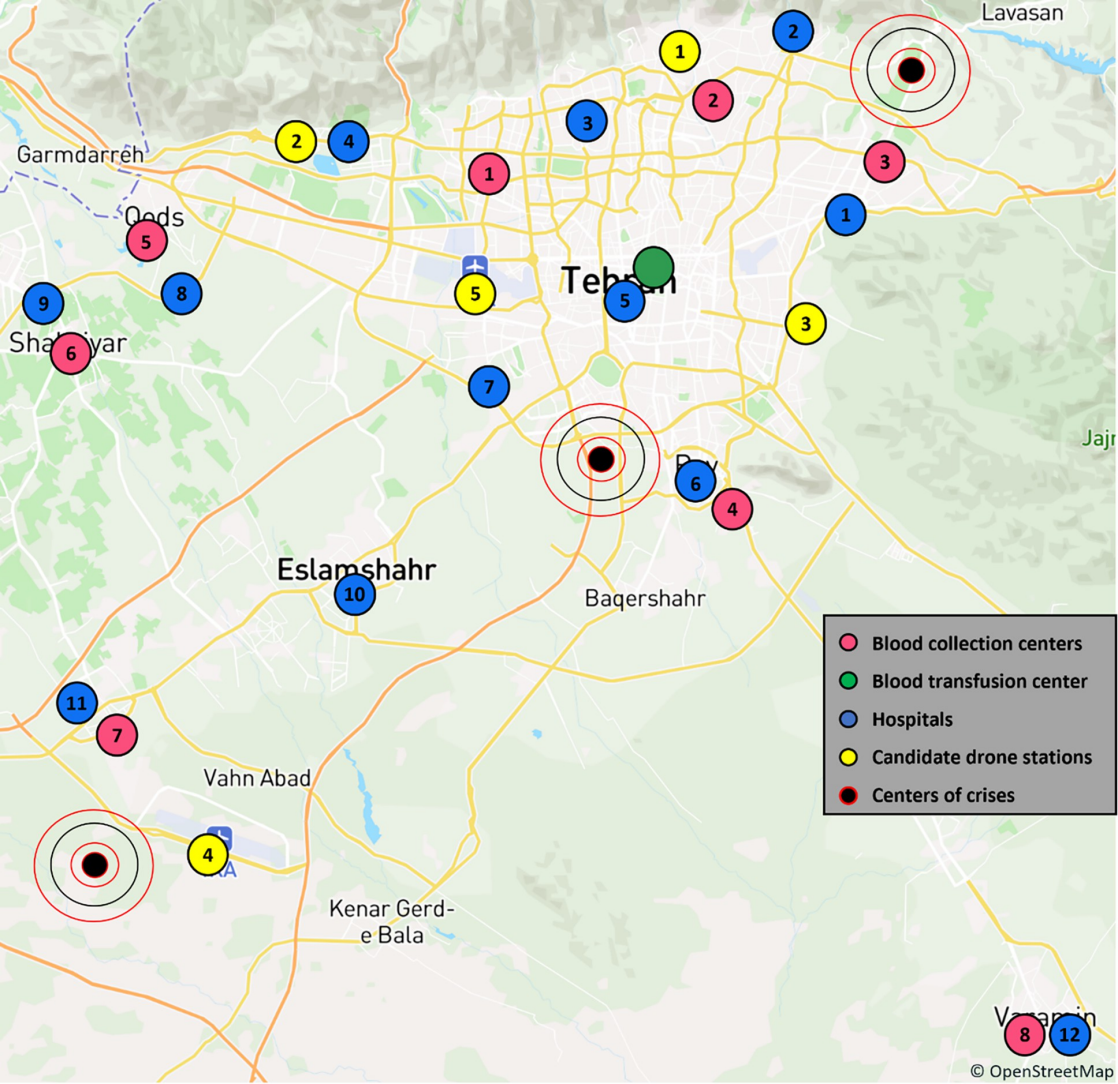
The position of BCCs, BTCs, hospitals, candidate drone stations and crisis centers. The foundational information and mapping data originate from OpenStreetMap and the OpenStreetMap Foundation.

A scenario-based approach allows the proposed model to be applied in a variety of possible scenarios. Crisis situations such as earthquakes are one of the most common scenarios to be considered in blood supply chain studies, among the scenarios that can be considered. In such conditions, blood demand increases, while on the other hand, having a fast and efficient transportation system is of particular importance. As can be seen in (Fig 4), in this study, three scenarios were considered based on the occurrence of a crisis in three parts of Tehran province: 1) occurrence of a crisis in the northern part of Tehran with a 50% probability, 2) occurrence of a crisis in the central part of Tehran with a 30% probability, and 3) occurrence of a crisis in the southern part of Tehran with a 20% probability. Vesal center, which is both a BCC and a BTC, was only investigated as a BTC in this study. Of the remaining BCCs, eight were investigated as BCCs, with the highest coverage at the provincial level. Moreover, twelve hospitals in this province have been selected in such a way that they not only cover the highly populated areas of the province in proportion, but are also within the range of at least one of the drones under investigation.

In this study, as previously mentioned, fleet selection is one of the decisions that must be made at the strategic level. Based on the information provided in the article by Otero et al. [35], three drones, M2 Matternet, Wingcopter 178, and Wingcopter 178 HL, have been considered as candidate drones for the network, and their information is presented in Table 4. Based on this information and the approximation provided by D’Andrea [61], shown in Eqs (37) and (38), the cost of flying a drone full of cargo per kilometer, as well as the cost of flying an empty drone per kilometer, has been calculated. These costs are also reported in the last two rows of Table 4.

**Table 4:** Costs and characteristics related to drones [35].

			Value	
Parameter	Symbol	M2 Matternet	Wingcopter 178	Wingcopter 178 HL
**Vehicle parameters**				
Maximum vehicle range (km)	*A*	25	100	45
Vehicle cruise velocity (km/hour)	*v*	36	40	40
Vehicle mass (kg)	*m*_*v*_	9.5	9.1	14
Vehicle payload capacity (kg)	*m*_*p*_	2	2	6
Vehicle cost (£)	*c*_*v*_	10,000	15,000	20,000
**Battery parameters**				
Battery cost (£/kWh)	*c*_*b*_	240		
Battery expected life (cycles)	*l*	300		
Power transfer efficiency (-)	*η*	0.5		
Lift-to-drag ratio (-)	*r*	3		
Power consumed by electronics (kWh)	*P*	0.1		
Charging efficiency (-)	*E*	0.8		
Electricity cost (£/kWh)	*c*_*e*_	0.15		
The cost of an empty drone flight (£/km)	*α*	0.019646	0.01866	0.027379
The cost of flying a drone full of goods (£/km)	*β*	0.023205	0.022219	0.038054


$$\beta =\left(\frac{c_{b}}{l}+\frac{c_{e}}{E}\right)\times \left(\frac{m_{\nu }+m_{p}}{370\eta r}+\frac{P}{\nu }\right)$$ (37)

$$\alpha =\left(\frac{c_{b}}{l}+\frac{c_{e}}{E}\right)\times \left(\frac{m_{\nu }}{370\eta r}+\frac{P}{\nu }\right)$$ (38)

Based on (Fig 4), Tables 5 and 6, It is important to note that the selection of all the places was made in consideration of the majority of the area and the demand of the province, and the occurrence of a crisis affects the performance of the BCCs and hospital demand. A distance between the mentioned centers was calculated based on the geographical coordinates. Dhote & Limbourg [34] calculated the cost of setting up a drone station, including rental costs, rental costs insurance, electricity (excluding recharging drones), staff not dedicated to the activity (maintenance, security, and training), and depreciation of equipment (5 years). In this study, as shown in (Fig 4), five candidate locations for drone stations were considered to be located either at helicopter pads or main airports in Tehran province. The establishment costs for each of these stations were calculated based on the Dhote & Limbourg [34] article and reported in Table 7.

**Table 5:** The number of required flights under scenario *θ* per day (M2 Matternet and Wingcopter 178).

*i*	BCCs	BTC	Scenario#1	Scenario #2	Scenario #3	*l*	BTC	Hospitals	Scenario #1	Scenario #2	Scenario #3
1	Sattari	Vesal	21	21	31	1	Vesal	Arash	119	84	48
2	Sadr	Vesal	14	28	40	2	Vesal	Masih daneshvari	130	67	47
3	Tehran pars	Vesal	16	19	41	3	Vesal	Erfan	138	71	51
4	Rey	Vesal	28	13	25	4	Vesal	Treata	90	63	85
5	Ghods	Vesal	32	35	21	5	Vesal	Loghman	82	127	58
6	Shahryaar	Vesal	36	42	15	6	Vesal	Firooz abadi	56	141	59
7	Robatkarim	Vesal	44	36	12	7	Vesal	Yaftabad	96	115	80
8	Varaamin	Vesal	50	49	50	8	Vesal	Ghods	56	55	96
						9	Vesal	Noor	44	37	115
						10	Vesal	Imam Reza	50	84	128
						11	Vesal	Robatkarim	49	47	105
						12	Vesal	Mofatteh	44	36	42

Underlined: Centers located in Tehran city (Tehran province includes Tehran city and several other cities)

**Table 6:** The number of required flights under scenario θ per day (Wingcopter 178 HL).

*i*	BCCs	BTC	Scenario #1	Scenario #2	Scenario #3	*l*	BTC	Hospitals	Scenario #1	Scenario #2	Scenario #3
1	Sattari	Vesal	7	7	11	1	Vesal	Arash	40	28	16
2	Sadr	Vesal	5	10	14	2	Vesal	Masih daneshvari	44	23	16
3	Tehran pars	Vesal	6	7	14	3	Vesal	Erfan	46	24	17
4	Rey	Vesal	10	5	9	4	Vesal	Treata	30	21	29
5	Ghods	Vesal	11	12	7	5	Vesal	Loghman	28	43	20
6	Shahryaar	Vesal	12	14	5	6	Vesal	Firooz abadi	19	47	20
7	Robatkarim	Vesal	15	12	4	7	Vesal	Yaftabad	32	39	27
8	Varaamin	Vesal	17	17	17	8	Vesal	Ghods	19	19	32
						9	Vesal	Noor	15	13	39
						10	Vesal	Imam Reza	17	28	43
						11	Vesal	Robatkarim	17	16	35
						12	Vesal	Mofatteh	15	12	14

Underlined: Centers located in Tehran city (Tehran province includes Tehran city and several other cities)

**Table 7:** The daily cost of setting up a drone station.

*j*	Drone stations	Set up cost (£)
1	North side helipad	166
2	West side helipad	156
3	East side helipad	147
4	Imam Khomeini International Airport	128
5	Mehrabad International Airport	137

Underlined: Centers located in Tehran city (Tehran province includes Tehran city and several other cities)

**Fig 5:**
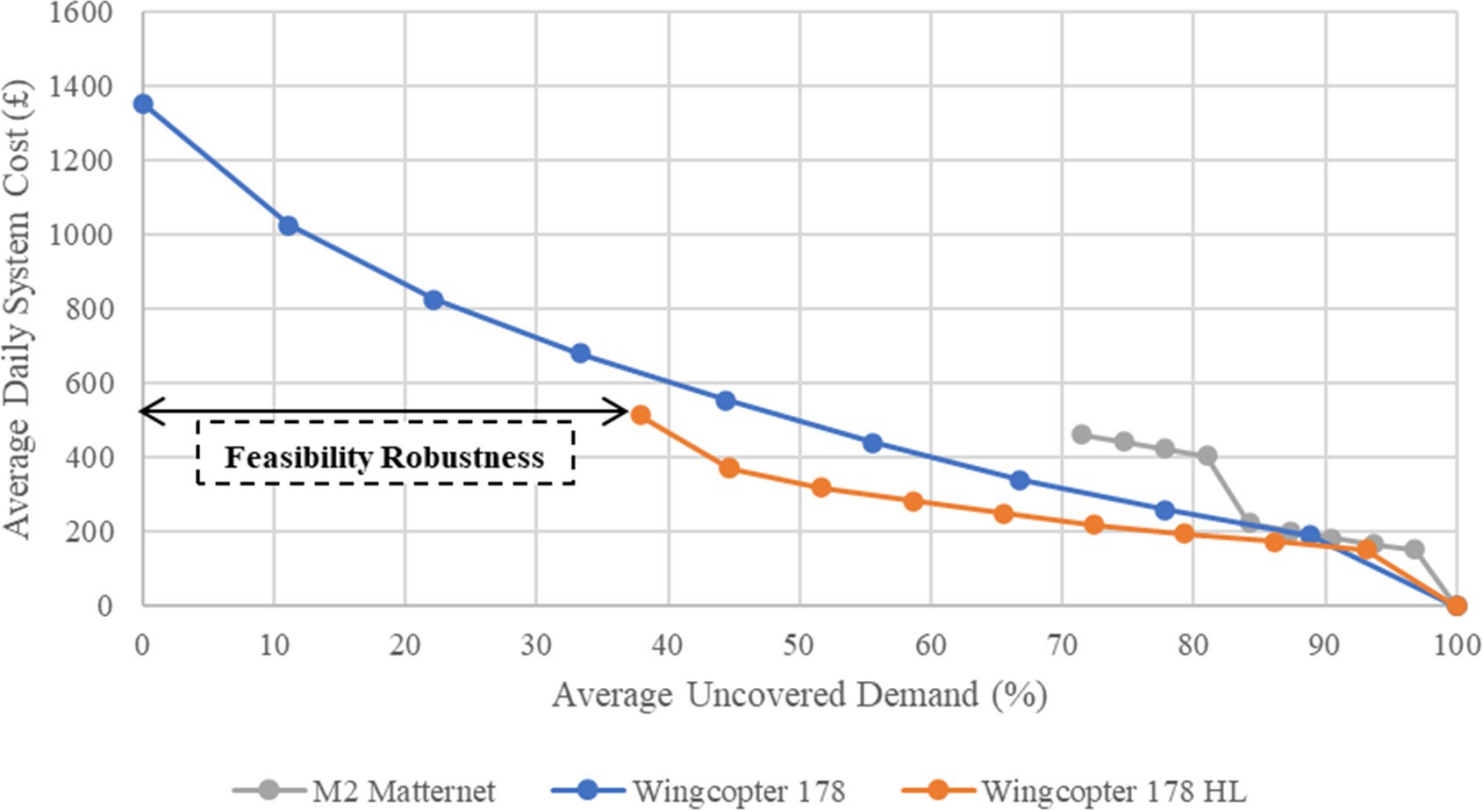
Pareto front for three candidate drones.

As shown in (Fig 5), by selecting ten points from the Pareto front, not only does it illustrate the trade-off between conflicting objectives, but it also provides a sensitivity analysis of the status of each drone. In light of this analysis, the use of the M2 Matternet drone is not recommended since it has a limited flight range and can cover only 28.5% of the system demand on average, and in this coverage range, two other drones have proven to be more cost-effective. While the Wingcopter 178HL drone had higher operational costs, it had the best cost-effective performance up to its maximum coverage capacity of 62.1%. Since it has a larger capacity and therefore requires half as many trips as the other two drones, the Wingcopter 178HL drone is the most cost-effective drone. On the other hand, because of its high flight range, the Wingcopter 178 drone was able to cover all the demand on average. Therefore, if the decision-maker wants to cover a network flow of up to 62.1%, Wingcopter 178HL is the best choice, but if they wish to cover a network flow greater than 62.1%, Wingcopter 178 will be the only choice. (Fig 5) illustrates the Pareto front, representing a set of optimal solutions. Within this front, the decision-maker enjoys the flexibility to select any point based on their individual preferences and priorities. It can be seen from each of the three charts that with an increase in the average daily cost of the system, the average uncovered demand decreases and vice versa. This indicates a trade-off between objective functions.

In order to present the results of the model, it is assumed that the Wingcopter 178HL drone has been selected for the blood transportation network. The model output for this drone is reported in Table 8. As mentioned, due to the 45-kilometer flight range of this drone, it was not possible to fully cover the demand, and therefore, at its maximum coverage, two drone stations were established on the east side of Tehran and Mehrabad Airport. Both of these locations were selected due to their proximity to the northern region (Due to the 50% chance of a crisis) while maintaining the centrality of the province, as well as their lower cost as compared to other candidate locations. On the other hand, focusing on the two terms of the cost objective function, namely drone operations and establishment costs, can demonstrate the intelligence of the model. In the low percentage of demand coverage, the model suggested establishing a drone station in Mehrabad, which is more affordable and central than establishing a drone station in the eastern part of Tehran. By changing route allocation and planning, the model has tried to avoid building expensive stations as much as possible, thereby increasing the cost of drone operations to cover a larger percentage of the population. In general, as expected, due to the volume and number of travel requests in Tehran city compared to other cities in Tehran province, the coverage of hospitals outside Tehran city has been entrusted to the non-drone transportation system (current).

**Table 8:** Results of the mathematical model for strategic planning (Wingcopter 178HL).

Optimal Solutions on pareto front	Average Daily System Cost (£)			Average Uncovered Demand	Stablished Drone Stations	Scenario #1		Scenario #2		Scenario #3	
	Drones Operation	Establishment	Total			BCC	Hospital	BCC	Hospital	BCC	Hospital
1	0	0	0	100%	-	-	-	-	-	-	-
2	16.674	137	153.674	93.1%	5	-	5	1	5	-	-
3	36.064	137	173.064	86.2%	5	1	5	-	5,7	-	3,5,7
4	58.408	137	195.408	79.3%	5	1,2	5,7	1	3,5,7	-	3,5,7
5	81.614	137	218.614	72.4%	5	1,2	3,5,7	1	3,5,7	1,2	3,5,7
6	111.996	137	248.996	65.5%	5	1,2	3,5,7	1,2	3,5,6,7	1,2	3,4,5,6,7
7	145.120	137	282.210	58.6%	5	1,2	3,4,5,6,7	1,2,4	3,5,6,7	1,2,4	3,4,5,6,7
8	181.133	137	318.133	51.7%	5	1,2	1,3,4,5,6,7	1,2,4	3,4,5,6,7	1,2	1,3,4,5,6,7
9	223.887	147	370.887	44.8%	3	1,2,3,4	1,2,3,5,6,7	1,2,3,4	1,2,3,5,6,7	1,2,3,4	1,2,3,5,6,7
10	230.497	284	514.497	37.9%	3	2,3,4	1,2,6	2,3,4	1,2,6	2,3,4	1,2,6
					5	1	3,4,5,7	1	3,4,5,7	1	3,4,5,7

Underlined: It is assumed that this solution is chosen from the Pareto front by the decision maker.

The use of a Scenario-based Robust Bi-objective Optimization approach along with the augmented ε-constraint method has created the condition for the decision maker to easily choose his desired decision according to his preferences, which is difficult to achieve using a scenario-based robust optimization approach. As shown in (Fig 5) and Table 8, the pareto solutions were produced by giving priority to each of the two objectives of average cost and average uncovered demand (feasibility robustness). The demonstration of the Feasibility Robustness of the proposed model can be observed in the gap between the minimum percentage of uncovered demand for the Wingcopter 178 HL with full coverage in (Fig 5). In contrast to the two-stage stochastic programming model, the proposed model allows for violations of constraints and controls the extent of these violations by achieving equilibrium with the cost objective function. However, the two-stage stochastic programming model is only feasible when using the Wingcopter 178, as shown in (Fig 5), since it is the only drone that can cover 100% of the demand without violating the constraints. Therefore, this supports the claim that the proposed model has higher efficiency and comprehensiveness for strategic planning.

As mentioned earlier, the proposed model in this study is a scenario-based model with a formulation in the form of Model (4). The deterministic form of this model, namely Model (39), has been considered in such a way that it can also allow for violating constraints, and the only difference between this model and the proposed model is its non-stochastic nature.

$$\begin{array}{l} Min\;z\\ Min\;\varepsilon \\ s.t.\\ Ax+Ty\geq b-\varepsilon \\ x,y,\varepsilon \geq 0 \end{array}$$ (39)

**Fig 6:**
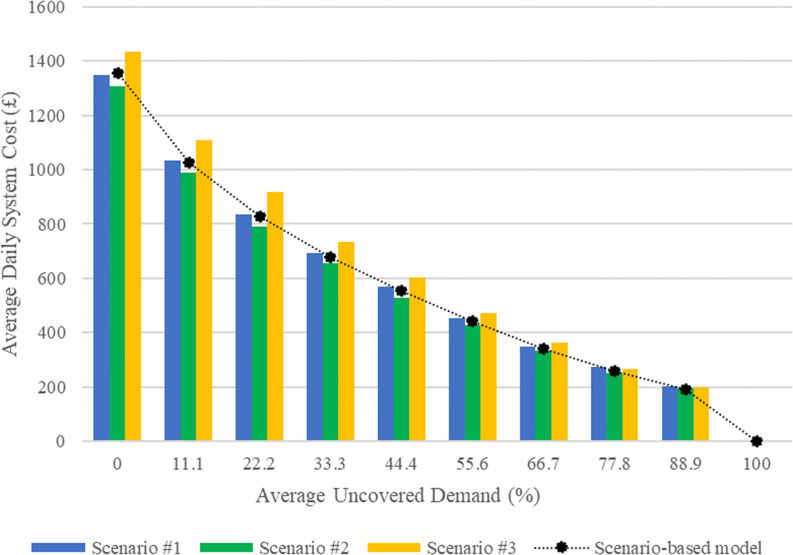
Pareto front to compare the performance of the deterministic model versus the scenario-based model (Wingcopter 178).

In order to validate the proposed model from the perspective of Optimality Robustness, both the proposed model and the deterministic model were investigated in a situation where the Wingcopter 178 drone is intended to be used. The deterministic model has been solved under the realization of each scenario, and the average daily cost of the system according to the solution provided by the model is also reported in (Fig 6). As can be seen from the comparison of the Pareto fronts in (Fig 6), the scenario-based model presented here is robust enough to handle changes in scenarios (occurrence of uncertainties).

To evaluate the robustness of a network design, one criterion is to consider the cost saved due to its robustness. Table 9 reports that the scenario-based model offers robust decisions to the decision maker based on their preferences, except when the drone system is not used. This leads to daily cost savings in comparison to decisions provided by the deterministic model when designing the network.

**Table 9:** Results of the deterministic model versus the scenario-based model (Wingcopter 178).

Optimal Solutions on pareto front	Average Uncovered Demand	Deterministic model			Scenario-based model	Cost saving (£)
		Scenario #1	Scenario #2	Scenario #3		
1	100%	0	0	0	0	0
2	88.9%	201.416	194.32	197.812	190.637	21.637
3	77.8%	272.381	250.719	264.771	258.556	12.203
4	66.7%	346.887	333.845	365.31	339.855	26.477
5	55.6%	453.491	425.431	471.738	441.175	27.135
6	44.4%	568.274	527.291	603.242	553.988	36.843
7	33.3%	693.103	655.771	735.537	679.967	44.51
8	22.2%	835.8	791.778	917.617	826.243	66.466
9	11.1%	1033.973	988.89	1107.521	1025.213	54.745
10	0%	1350.38	1307.971	1435.123	1354.606	29.656

### 4.2. Operational planning

**Fig 7:**
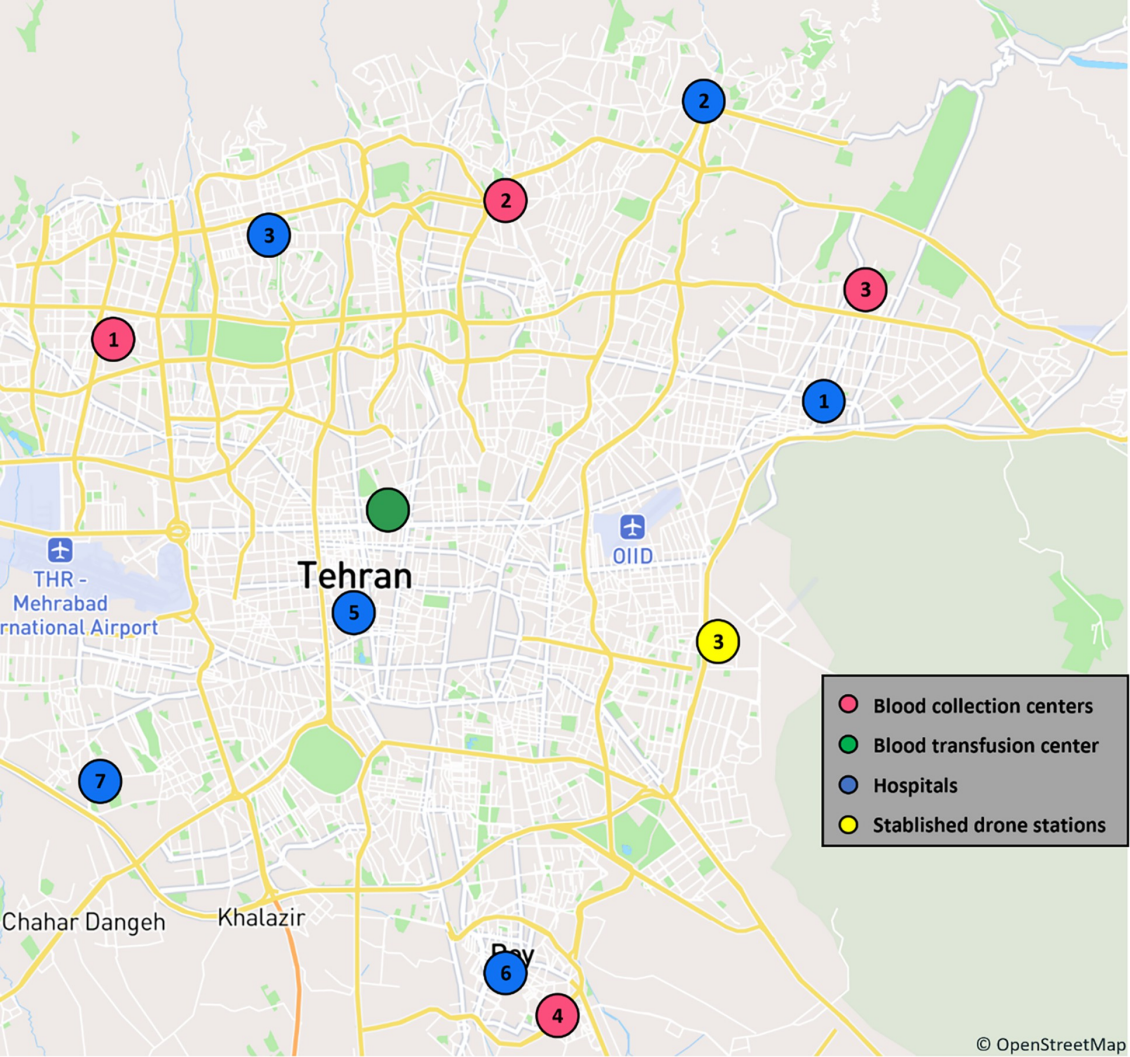
The network related to the solution chosen by the decision maker. The foundational information and mapping data originate from OpenStreetMap and the OpenStreetMap Foundation.

In operational planning, it is assumed that the decision maker at the strategic level has chosen the solution specified in Table 8, and the network of this solution is shown in (Fig 7). Moreover, as shown in Tables 10 and 11, in both layers of the transportation network, the number of flights is assumed to follow a uniform distribution between one and three, and the deadlines for reaching each BCC and hospital are also assumed to follow the same distribution between twenty minutes and sixty minutes. It should be noted that each node is assumed to have a zero-service time.

**Table 10:** Number of required flights from BCC/ BTC point to BTC/Hospital point (Wingcopter 178 HL).

*i*	BCCs	BTC	# Of required flights	*l*	BTC	Hospitals	# Of required flights
1	Sattari	Vesal	1	1	Vesal	Arash	3
2	Sadr	Vesal	2	2	Vesal	Masih daneshvari	1
3	Tehran pars	Vesal	2	3	Vesal	Erfan	2
4	Rey	Vesal	2	5	Vesal	Loghman	1
				6	Vesal	Firooz abadi	2
				7	Vesal	Yaftabad	1

**Table 11:** Deadline for traveling to the BCC/hospital point (minutes).

*i*	BCCs	Deadlines (minutes)	*l*	Hospitals	Deadlines (minutes)
1	Sattari	42	1	Arash	36
2	Sadr	58	2	Masih daneshvari	58
3	Tehran pars	46	3	Erfan	27
4	Rey	36	5	Loghman	24
			6	Firooz abadi	31
			7	Yaftabad	30

Assuming in the operational model that the fleet’s cost only includes the cost of purchasing the drone, Eq (40) is proposed for calculating it.

$$\mathrm{Total}\;\mathrm{cost}\;\mathrm{of}\;\mathrm{the}\;\mathrm{fleet}=c_{v}\sum_{j\in V^{\prime }}\sum_{u\in U}x_{1j}^{u1}$$ (40)

**Fig 8:**
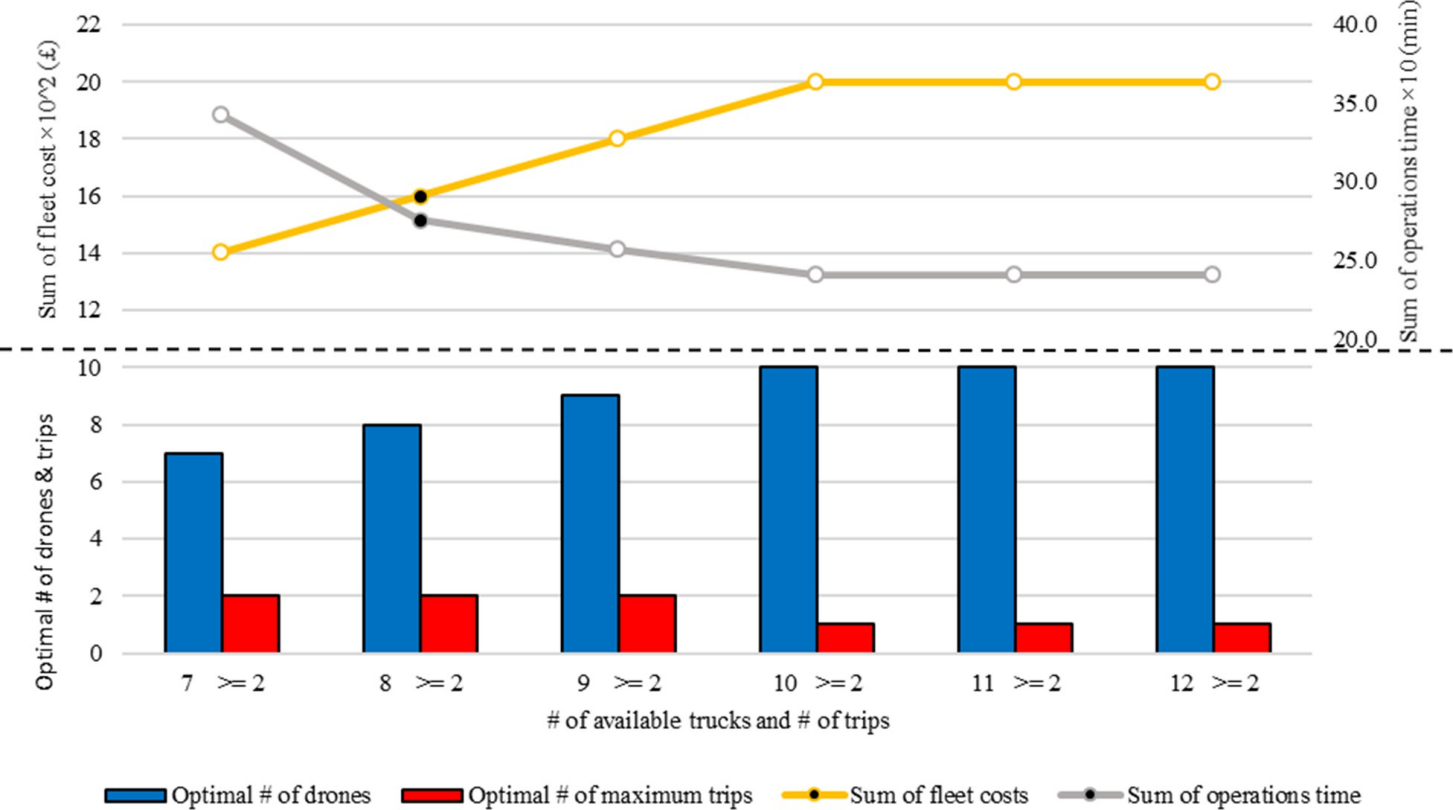
Fleets & time related graphical results of the mathematical programming model for operational planning (Wingcopter 178HL).

As shown in Table 12, considering the conditions of the case study, if one to six drones with the ability to make any number of trips are considered for the transportation network, a feasible solution to the problem will not be found. These conditions continue for seven, eight, and nine drones with the ability to make only one trip. However, if the travel capability in any of these cases is increased to two or more, the drones can satisfy all the constraints, and the model’s situation becomes feasible. It is obvious that when nine drones are used in the system, the operation time is less than in the case where 8 drones are used, despite the higher fleet cost. The graph of the trend of the change in the sum of operations time versus the change in the total fleet cost, shown in (Fig 8), clearly demonstrates this fact. But another point that can be taken from this figure is to fix the sum of operations time and the total cost of the fleet from one place to the next. That is, when the number of drones has changed from ten to eleven under equal conditions, the model still recommends the use of ten drones. This is also indicative of the intelligence of the model, in that when the problem conditions are such that the model sees that deploying another drone will not improve the sum of operations time, the model recommends using the minimum number of drones possible. This is the reason why in Table 12, when eight drones are considered for the system and each can fly, for example, five times, the model suggests only two flights.

**Table 12:** Fleets & time related results of the mathematical programming model for operational planning (Wingcopter 178HL).

# Of available drones	# Of trips allowed	# Of used drones	Maximum # Of trips made	sum of operations time (minutes)	Sum fleet cost (£)
1	> = 1	-	-	infeasible	-
2	> = 1	-	-	infeasible	-
3	> = 1	-	-	infeasible	-
4	> = 1	-	-	infeasible	-
5	> = 1	-	-	infeasible	-
6	> = 1	-	-	infeasible	-
7	> = 2	7	2	342.7981	140,000
8	> = 2	8	2	275.4901	160,000
9	> = 2	9	2	256.4051	180,000
10	> = 1	10	1	240.7551	200,000
11	> = 1	10	1	240.7551	200,000
12	> = 1	10	1	240.7551	200,000

**Fig 9:**
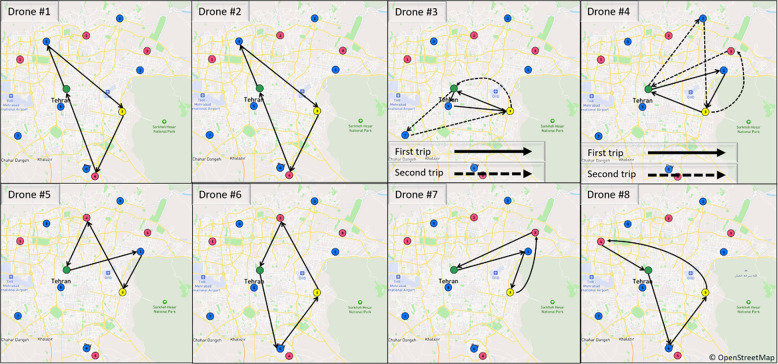
Routing related results of the mathematical programming model for operational planning (Wingcopter 178HL). The foundational information and mapping data originate from OpenStreetMap and the OpenStreetMap Foundation.

**Fig 10:**
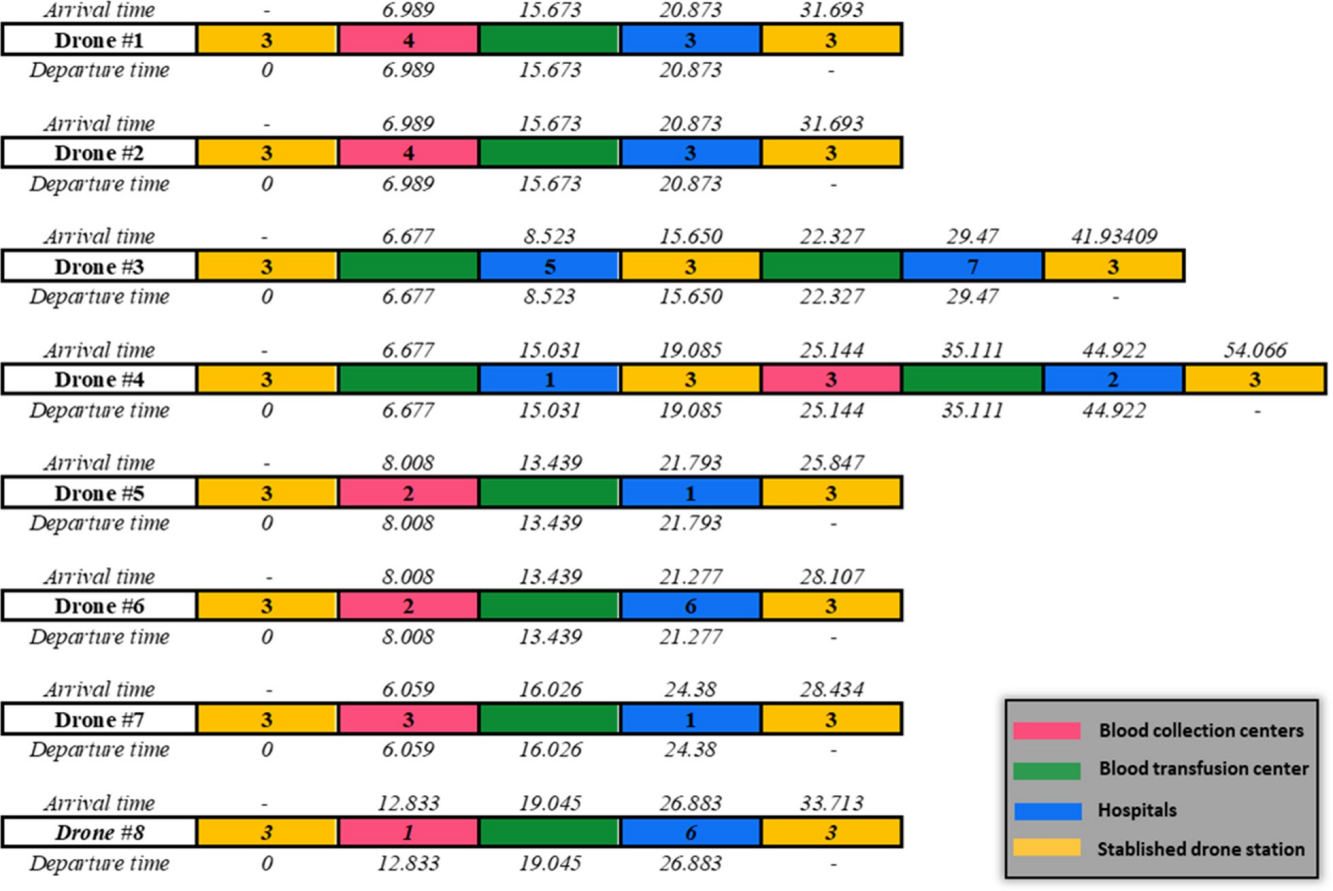
Scheduling related results of the mathematical programming model for operational planning (Wingcopter 178HL).

As mentioned earlier, in the mathematical programming model presented for planning at the operational level, routing and scheduling are the two main decisions of the model as outputs. Therefore, the schematic of this output for the case where eight drones are considered with the possibility of making two trips for each drone, is shown in Figs 9 and 10, which are related to routing and scheduling, respectively. By comparing the outputs to Tables 10 and 11, it can be seen that the transportation system has been operationally planned in such a way that all deliveries have taken place in the designated time window. As shown in (Fig 9), the mathematical programming model suggests that two drones #3 and #4 make two trips and the rest of the drones make only one trip. For example, drone #4 performs two operations or trips, which are usually referred to as tours in the routing literature, as follows:

The first trip: the drone takes off empty from the east drone station at the beginning of the planning horizon. This drone arrived at Vesal center at 6.677 and since the service time is 0, at 6.677, it moved towards it by taking the blood products needed by Arash Hospital. It will return to the eastern drone station to recharge its batteries after arriving in less time than the hospital’s time window and delivering blood products.

The second trip: the drone departed from the drone station at 19.085 and arrived at Tehran Pars BCC at 25.144. Then, from there, the collected blood will be transported to the vesal center, and from there, after unloading, it will be loaded with the blood products required by Masih Daneshvari Hospital and will move towards this hospital, and finally, at the time of 54.066, it will return to the drone station again.

This section provided analysis and managerial insights that could be applied to the strategic and operational planning for the use of drones in the urban blood transportation system. In order to use the presented hierarchical model more efficiently, it is suggested that based on the provided managerial insights, the analysis that was done to select the drone used in the network should be carried out and then the appropriate drone should be selected based on the preferences. After making decisions related to network design, the presented operational model could be used for short-term operational planning, taking into account the network conditions and deadlines in the short-term planning horizon. Additionally, it was suggested to implement the operational model on the simulated data to determine the number of drones required in each drone station.

## 5. Conclusions

In conclusion, this research presents an advancement in the field of logistics and mathematical programming by introducing two models for blood transportation via drones in urban areas. These models, namely the strategic planning approach and the operational planning model, offer decision-makers a comprehensive framework for addressing location-allocation, fleet selection, fleet allocation, fleet sizing, and routing-scheduling decisions. Through rigorous validation in crisis scenarios, the effectiveness of these models in overcoming challenges related to urban blood transportation is evident. The cost-effectiveness and operational capabilities of different drone models have been demonstrated, aiding in the optimization of operational efficiency and cost reduction. Additionally, minimizing the number of drones has been shown to enhance operational time, efficiency, and cost savings.

The practical application of these models in real-life situations underlines their potential benefits in healthcare and logistics. The hierarchical model’s adaptability to similar supply chain networks in different urban contexts encourages further exploration. The evolving complexity of scenarios underscores the need for alternative methodologies such as sample average approximation (SAA) or L-shaped with augmented ε-constraint approach. For larger-scale problems, the integration of metaheuristic and heuristic algorithms is recommended due to inherent computational challenges.

In summary, this research not only advances the field of mathematical optimization but also presents a decision-making framework for the implementation of drone transportation systems within urban blood supply chains. Looking ahead, there are several promising directions for future research. These include exploring task assignment using queueing theory, fostering interdisciplinary collaboration across diverse domains, and finding the right balance between automation and human involvement. Additionally, further investigations could incorporate factors such as meteorological conditions, environmental considerations, and socioeconomic impacts to enhance the effectiveness and sustainability of drone-based blood transportation systems. The emerging realm of drone logistics, coupled with the potential synergies between drone logistics and blood transportation, offers exciting opportunities for researchers to pioneer innovative approaches. As this field continues to develop, it holds the potential to revolutionize urban blood transportation, optimize operational efficiency, and ensure the timely delivery of vital medical resources. As demonstrated in this study, the integration of drones into existing transportation and logistics frameworks could potentially yield transformative outcomes, particularly within the realm of e-commerce logistics.

## References

[1] Ozkan O. Multi‐objective optimization of transporting blood products by routing UAVs: the case of Istanbul. Int Trans Oper Res. 2023 Jan 7;30(1):302–27.

[2] WHO. Blood safety and availability [Internet]. 2022. Available from: https://www.who.int/news-room/fact-sheets/detail/blood-safety-and-availability

[3] Amukele T, Ness PM, Tobian AAR, Boyd J, Street J. Drone transportation of blood products. Transfusion. 2017 Mar;57(3):582–8. doi: 10.1111/trf.13900 27861967

[4] Farrokhizadeh E, Seyfi-Shishavan SA, Satoglu SI. Blood supply planning during natural disasters under uncertainty: a novel bi-objective model and an application for red crescent. Ann Oper Res. 2021 Feb 22;

[5] Thiels CA, Aho JM, Zietlow SP, Jenkins DH. Use of unmanned aerial vehicles for medical product transport. Vol. 34, Air Medical Journal. 2015. p. 104–8. doi: 10.1016/j.amj.2014.10.011 25733117

[6] Rosser JC, Vignesh V, Terwilliger BA, Parker BC. Surgical and Medical Applications of Drones: A Comprehensive Review. JSLS J Soc Laparoendosc Surg. 2018;22(3):e2018.00018. doi: 10.4293/JSLS.2018.00018 30356360 PMC6174005

[7] Oakey A, Grote M, Smith A, Cherrett T, Pilko A, Dickinson J, et al. Integrating drones into NHS patient diagnostic logistics systems: Flight or fantasy? PLoS One. 2022 Dec 22;17(12):e0264669. doi: 10.1371/journal.pone.0264669 36548251 PMC9778562

[8] Stephan F, Reinsperger N, Grünthal M, Paulicke D, Jahn P. Human drone interaction in delivery of medical supplies: A scoping review of experimental studies. PLoS One. 2022 Apr 28;17(4):e0267664. doi: 10.1371/journal.pone.0267664 35482656 PMC9049298

[9] Kaplan D. How medical drones help save lives in Tanzania [Internet]. 2019 [cited 2023 Jun 5]. Available from: https://www.dhl.com/global-en/delivered/insights/medical-drones-save-lives-tanzania.html

[10] Kolodny L. Drones will deliver defibrillators to 911 callers to help treat cardiac arrest [Internet]. 2017 [cited 2023 Jun 5]. Available from: https://www.cnbc.com/2017/10/10/watch-flirtey-and-remsa-deliver-defibrillators-by-drone-to-911-callers.html

[11] Peters A. Switzerland Is Getting A Network Of Medical Delivery Drones [Internet]. 2017 [cited 2023 Jun 5]. Available from: https://www.fastcompany.com/40467761/switzerland-is-getting-a-network-of-medical-delivery-drones

[12] Ling G, Draghic N. Aerial drones for blood delivery. Transfusion. 2019 Apr 13;59(S2):1608–11. doi: 10.1111/trf.15195 30980745

[13] Nisingizwe MP, Ndishimye P, Swaibu K, Nshimiyimana L, Karame P, Dushimiyimana V, et al. Effect of unmanned aerial vehicle (drone) delivery on blood product delivery time and wastage in Rwanda: a retrospective, cross-sectional study and time series analysis. Lancet Glob Heal. 2022 Apr;10(4):e564–9. doi: 10.1016/S2214-109X(22)00048-1 35303465

[14] Moshref-Javadi M, Winkenbach M. Applications and Research avenues for drone-based models in logistics: A classification and review. Expert Syst Appl. 2021 Sep;177:114854.

[15] Amukele TK, Sokoll LJ, Pepper D, Howard DP, Street J. Can Unmanned Aerial Systems (Drones) Be Used for the Routine Transport of Chemistry, Hematology, and Coagulation Laboratory Specimens? PLoS One. 2015 Jul 29;10(7):e0134020. doi: 10.1371/journal.pone.0134020 26222261 PMC4519103

[16] Benayad A, Malasse O, Belhadaoui H, Benayad N. Unmanned Aerial Vehicle in the Logistics of Pandemic Vaccination: An Exact Analytical Approach for Any Number of Vaccination Centres. Healthcare. 2022 Oct 20;10(10):2102. doi: 10.3390/healthcare10102102 36292549 PMC9602990

[17] Beliën J, Forcé H. Supply chain management of blood products: A literature review. Eur J Oper Res. 2012 Feb;217(1):1–16.

[18] Nahmias S. Perishable Inventory Theory: A Review. Oper Res. 1982 Aug;30(4):680–708. doi: 10.1287/opre.30.4.680 10298625

[19] Osorio AF, Brailsford SC, Smith HK. A structured review of quantitative models in the blood supply chain: a taxonomic framework for decision-making. Int J Prod Res. 2015 Dec 17;53(24):7191–212.

[20] Rais A, Viana A. Operations Research in Healthcare: a survey. Int Trans Oper Res. 2011 Jan;18(1):1–31.

[21] Prastacos GP. Blood Inventory Management: An Overview of Theory and Practice. Manage Sci. 1984 Jul;30(7):777–800.

[22] Kim S, Pasupathy R, Henderson SG. A Guide to Sample Average Approximation. In 2015. p. 207–43.

[23] Laporte G, Louveaux F V. The integer L-shaped method for stochastic integer programs with complete recourse. Oper Res Lett. 1993 Apr;13(3):133–42.

[24] Kim SJ, Lim GJ, Cho J, Côté MJ. Drone-Aided Healthcare Services for Patients with Chronic Diseases in Rural Areas. J Intell Robot Syst. 2017 Oct 4;88(1):163–80.

[25] Chauhan D, Unnikrishnan A, Figliozzi M. Maximum coverage capacitated facility location problem with range constrained drones. Transp Res Part C Emerg Technol. 2019 Feb;99.

[26] Ozkan O, Atli O. Transporting COVID-19 testing specimens by routing unmanned aerial vehicles with range and payload constraints: the case of Istanbul. Transp Lett. 2021 May 28;13(5–6).

[27] Ghelichi Z, Gentili M, Mirchandani PB. Logistics for a fleet of drones for medical item delivery: A case study for Louisville, KY. Comput Oper Res. 2021 Nov;135:105443.

[28] Jung H, Kim J. Drone scheduling model for delivering small parcels to remote islands considering wind direction and speed. Comput Ind Eng. 2022 Jan;163:107784.

[29] Ghelichi Z, Gentili M, Mirchandani PB. Drone logistics for uncertain demand of disaster-impacted populations. Transp Res Part C Emerg Technol. 2022 Aug;141:103735.

[30] Rajesh Chauhan D, Unnikrishnan A, Figliozzi MA, Boyles SD. Robust Multi-Period Maximum Coverage Drone Facility Location Problem Considering Coverage Reliability. Transp Res Rec J Transp Res Board. 2022 Apr 21;036119812210872.

[31] Shi Y, Lin Y, Li B, Yi Man Li R. A bi-objective optimization model for the medical supplies’ simultaneous pickup and delivery with drones. Comput Ind Eng. 2022 Sep;171:108389. doi: 10.1016/j.cie.2022.108389 35791409 PMC9245375

[32] Park Y, Lee S, Sung I, Nielsen P, Moon I. Facility Location-Allocation Problem for Emergency Medical Service With Unmanned Aerial Vehicle. IEEE Trans Intell Transp Syst. 2022;1–15.

[33] Wen T, Zhang Z, Wong KKL. Multi-Objective Algorithm for Blood Supply via Unmanned Aerial Vehicles to the Wounded in an Emergency Situation. PLoS One. 2016 May 10;11(5):e0155176. doi: 10.1371/journal.pone.0155176 27163361 PMC4862655

[34] Dhote J, Limbourg S. Designing unmanned aerial vehicle networks for biological material transportation–The case of Brussels. Comput Ind Eng. 2020 Oct;148.

[35] Otero Arenzana A, Escribano Macias JJ, Angeloudis P. Design of Hospital Delivery Networks Using Unmanned Aerial Vehicles. Transp Res Rec J Transp Res Board. 2020 May 23;2674(5):405–18.

[36] Ghasemi S, Tavakkoli-Moghaddam R, Hamid M, Hosseinzadeh M. Sustainable Facility Location-Routing Problem for Blood Package Delivery by Drones with a Charging Station. In 2021. p. 3–14.

[37] Rashidzadeh E, Hadji Molana SM, Soltani R, Hafezalkotob A. Assessing the sustainability of using drone technology for last-mile delivery in a blood supply chain. J Model Manag. 2021 Nov 25;16(4):1376–402.

[38] Hou W, Fang T, Pei Z, He QC. Integrated design of unmanned aerial mobility network: A data-driven risk-averse approach. Int J Prod Econ. 2021 Jun;236.

[39] Al-Rabiaah S, Hosny M, AlMuhaideb S. A Greedy Heuristic Based on Optimizing Battery Consumption and Routing Distance for Transporting Blood Using Unmanned Aerial Vehicles. Electronics. 2022 Oct 20;11(20):3399.

[40] Kim D, Lee K, Moon I. Stochastic facility location model for drones considering uncertain flight distance. Ann Oper Res. 2019 Dec 13;283(1–2).

[41] Zhu T, Boyles SD, Unnikrishnan A. Two-stage robust facility location problem with drones. Transp Res Part C Emerg Technol. 2022 Apr;137:103563.

[42] Shavarani SM, Nejad MG, Rismanchian F, Izbirak G. Application of hierarchical facility location problem for optimization of a drone delivery system: a case study of Amazon prime air in the city of San Francisco. Int J Adv Manuf Technol. 2018 Apr 7;95(9–12).

[43] Rezaei Kallaj M, Hasannia Kolaee M, Mirzapour Al-e-hashem SMJ. Integrating bloodmobiles and drones in a post-disaster blood collection problem considering blood groups. Ann Oper Res. 2022 Aug 27;

[44] Rojas Viloria D, Solano‐Charris EL, Muñoz‐Villamizar A, Montoya‐Torres JR. Unmanned aerial vehicles/drones in vehicle routing problems: a literature review. Int Trans Oper Res. 2021 Jul 2;28(4):1626–57.

[45] Grippa P, Behrens DA, Wall F, Bettstetter C. Drone delivery systems: job assignment and dimensioning. Auton Robots. 2019 Feb 18;43(2):261–74.

[46] Xia Y, Zeng W, Xing X, Zhan Y, Tan KH, Kumar A. Joint optimisation of drone routing and battery wear for sustainable supply chain development: a mixed-integer programming model based on blockchain-enabled fleet sharing. Ann Oper Res. 2023 Aug 10;327(1):89–127.

[47] Liu C, Chen H, Li X, Liu Z. A scheduling decision support model for minimizing the number of drones with dynamic package arrivals and personalized deadlines. Expert Syst Appl. 2021 Apr;167:114157.

[48] Gómez-Lagos J, Rojas-Espinoza B, Candia-Véjar A. On a Pickup to Delivery Drone Routing Problem: Models and algorithms. Comput Ind Eng. 2022 Oct;172:108632.

[49] Yuan X, Zhu J, Li Y, Huang H, Wu M. An enhanced genetic algorithm for unmanned aerial vehicle logistics scheduling. IET Commun. 2021 Jun;15(10):1402–11.

[50] Moshref-Javadi M, Lee S, Winkenbach M. Design and evaluation of a multi-trip delivery model with truck and drones. Transp Res Part E Logist Transp Rev. 2020 Apr;136.

[51] Bazaraa MS, Jarvis JJ, Sherali HD. Linear Programming and Network Flows. Wiley; 2009.

[52] Govindan K, Fattahi M, Keyvanshokooh E. Supply chain network design under uncertainty: A comprehensive review and future research directions. Eur J Oper Res. 2017 Nov;263(1):108–41.

[53] Pishvaee MS MSBS. Biomass to Biofuel Supply Chain Design and Planning Under Uncertainty. Elsevier; 2021.

[54] Birge JR, Louveaux F. Introduction to Stochastic Programming. New York, NY: Springer New York; 2011.

[55] Mulvey JM, Vanderbei RJ, Zenios SA. Robust Optimization of Large-Scale Systems. Oper Res. 1995 Apr;43(2):264–81.

[56] Khoshabi P, Nejati E, Ahmadi SF, Chegini A, Makui A, Ghousi R. Developing a Multi-Criteria Decision Making approach to compare types of classroom furniture considering mismatches for anthropometric measures of university students. PLoS One. 2020 Sep 17;15(9):e0239297. doi: 10.1371/journal.pone.0239297 32941538 PMC7498002

[57] Sabripoor A, Amirsahami A, Ghousi R. Credibility based chance constrained programming for parallel machine scheduling under linear deterioration and learning effects with considering setup times dependent on past sequences. J Proj Manag. 2023;8(3):177–90.

[58] Torabzadeh SA, Nejati E, Aghsami A, Rabbani M. A dynamic multi-objective green supply chain network design for perishable products in uncertain environments, the coffee industry case study. Int J Manag Sci Eng Manag. 2022 Jul 3;17(3):220–37.

[59] Mavrotas G. Effective implementation of the ε-constraint method in Multi-Objective Mathematical Programming problems. Appl Math Comput. 2009 Jul;213(2):455–65.

[60] Iran Blood Transfusion Organization [Internet]. Available from: https://www.ibto.ir

[61] D’Andrea R. Guest Editorial Can Drones Deliver? IEEE Trans Autom Sci Eng. 2014 Jul;11(3):647–8.

